# Supplementary material for: Ethnic differences in current smoking and former smoking in the Netherlands and the contribution of socioeconomic factors: a cross-sectional analysis of the HELIUS study
Source: BMJ Open. 2017 Jul 10;7(7):e016041. doi: 10.1136/bmjopen-2017-016041 (PMC5541454; doi:10.1136/bmjopen-2017-016041)
Supplement: Author's manuscript [file bmjopen-2017-016041.draft_revisions.pdf]

BMJ Open

# Ethnic differences in smoking and former smoking in the Netherlands and the contribution of socio-economic factors: a cross-sectional analysis of the HELIUS Study


| | |
|---|---|
| Journal: | *BMJ Open* |
| Manuscript ID | bmjopen-2017-016041 |
| Article Type: | Research |
| Date Submitted by the Author: | 20-Jan-2017 |



| | |
|---|---|
| Complete List of Authors: | Brathwaite, Rachel; London School of Hygiene and Tropical Medicine, Non-communicable Disease Epidemiology<br>Smeeth, Liam; London School of Hygiene and Tropical Medicine, Epidemiology and Population Health<br>Addo, Juliet; London School of Hygiene and Tropical Medicine, Epidemiology and Population Health<br>Kunst, Anton; University of Amsterdam, Dept. of Public Health, Academic Medical Center (AMC)<br>Peters, Ron; Academic Medical Center, Cardiology<br>Snijder, Marieke; Academic Medical Centre, University of Amsterdam, Department of Public Health<br>Derks, Eske; AMC, Department of Psychiatry<br>Agyemang, Charles; Amsterdam Medical Center |



| | |
|---|---|
| <b>Primary Subject Heading</b>: | Smoking and tobacco |
| Secondary Subject Heading: | Smoking and tobacco |
| Keywords: | EPIDEMIOLOGY, PUBLIC HEALTH, STATISTICS & RESEARCH METHODS |
| | |


SCHOLARONE™
Manuscripts

## TITLE PAGE

**Title:** Ethnic differences in smoking and former smoking in the Netherlands and the contribution of socio-economic factors: a cross-sectional analysis of the HELIUS Study

**Corresponding Author:**

Rachel Brathwaite, MSc.

Department of Non-communicable Disease Epidemiology, London School of Hygiene and Tropical Medicine, London, WC1E7HT, UK.

E-mail: Rachel.Brathwaite@lshtm.ac.uk

Telephone: +44 (0) 2076368636 ext 2495

**Authors:**

Rachel Brathwaite MSc[1], Liam Smeeth PhD[1], Juliet Addo PhD[1], Anton E Kunst PhD[2], Ron JG Peters PhD[3], Marieke B Snijder PhD[2], Eske M Derks PhD[4], Charles Agyemang PhD[2]

[1]Department of Non-Communicable Disease Epidemiology, London School of Hygiene and Tropical Medicine, London, UK.

[2]Department of Public Health, Academic Medical Center, University of Amsterdam, Amsterdam, The Netherlands.

[3]Department of Cardiology, Academic Medical Center, University of Amsterdam, Amsterdam, The Netherlands.

[4]Department of Psychiatry, Academic Medical Center, Amsterdam, The Netherlands.

**Key words:** HELIUS study, smoking cessation, prevalence, ethnicity, socio-economic status (SES)

**Word count:** 3469



For peer review only - http://bmjopen.bmj.com/site/about/guidelines.xhtml

## ABSTRACT

**Objectives:** Data exploring how much of the ethnic differences in smoking prevalence and former smoking are explained by socioeconomic status (SES) are lacking. We therefore assessed ethnic differences in smoking prevalence and former smoking and the contribution of both educational level and occupational-related SES to the observed ethnic differences in smoking behaviour.

**Methods:** Data of 22,929 participants (aged 18-70 years) from the multi-ethnic cross-sectional HELIUS study in the Netherlands were analysed. Poisson regression models with a robust variance were used to estimate prevalence ratios.

**Results:** Compared to the Dutch, after adjustment for age and marital status, smoking prevalence was higher in men of Turkish (PR 1.69, 95% CI 1.54 to 1.86), African Surinamese (1.55, 1.41 to 1.69) and South-Asian Surinamese origin (1.53, 1.40 to 1.68), whereas among women, smoking prevalence was higher in Turkish, similar in African-Surinamese but lower in all other ethnic origin groups. All ethnic minority groups, except Ghanaians, had a significantly lower smoking cessation prevalence than the Dutch. Socio-economic gradients in smoking (higher prevalence among those lower educated and with lower level employment) were observed in all groups except Ghanaian women (a higher prevalence was observed in the higher educated). Ethnic differences in smoking prevalence and former smoking are largely, but not completely, explained by socioeconomic factors.

**Conclusions:** Our findings imply that we would be better able to reduce smoking in the Netherlands by factoring in ethnic diversity in smoking that we now better understand.



For peer review only - http://bmjopen.bmj.com/site/about/guidelines.xhtml

**Strengths and limitations**

- The HELIUS study utilized large sample sizes in each ethnic group and participants were recruited in a systematic manner to reflect a representative sample of adults in each ethnic group living in Amsterdam.[1]
- Smoking status may be underreported since biochemical methods were not used to verify self-reported smoking statuses.
- Smokers were not asked about the use of other tobacco products, so the use of tobacco might have not been fully captured in this study.
- Smoking cessation was assessed by using quit ratios, which is a cumulative measure of a number of former smokers in the ethnic group, which occurred over time and not necessarily an indicator of a recent smoking cessation or assessment at the individual level.

For peer review only



For peer review only - http://bmjopen.bmj.com/site/about/guidelines.xhtml

**WHAT IS ALREADY KNOWN ON THIS SUBJECT?**

In the Netherlands, ethnic differences in smoking has been observed among the Turkish, Surinamese and Moroccan ethnic minority groups with smoking being associated with most lower educated men and higher educated women. However, this was only investigated in the 35-60 age group, without distinguishing between African-Surinamese and South-Asian Surinamese and without including adults of Ghanaian descent, one of the largest ethnic minority groups in the Netherlands. Comparisons of both smoking prevalence and former smoking has not been compared to the Dutch host population and the extent to which educational and occupational levels explain ethnic differences in smoking and former smoking has not been studied.

**WHAT DOES THIS STUDY ADD?**

This study compared smoking prevalence and former smoking of four of the largest ethnic minority populations to the Dutch. The findings suggest that ethnic differences in smoking prevalence and former smoking in the Netherlands are largely but not completely explained by socio-economic differences. These findings imply that anti-smoking policies designed to target different ethnic groups and those of lower socio-economic status may substantially reduce smoking particularly among men in the Netherlands. However, among Ghanaian women, smoking prevention measures should particularly include higher educated women.

For peer review only



For peer review only - http://bmjopen.bmj.com/site/about/guidelines.xhtml

## INTRODUCTION

Ethnic differences in mortality rates and prevalence of lifestyle risk factors for diseases have been observed in the Netherlands.[2] Smoking is a leading lifestyle risk factor that accounts for premature morbidity and mortality from several diseases in populations worldwide.[3] Higher rates of smoking, as well as lower rates of former smoking are more present in disadvantaged than advantaged groups in society.[4-6] Most ethnic minority groups may be of lower socio-economic status (SES) than European host populations.[7] Evidence suggests variations in smoking prevalence across ethnic groups in high-income countries.[8,9] Differences in smoking prevalence among ethnic minority groups in European countries may be a reflection of their pre-migratory smoking status, or factors of the post-migratory environment, which influences smoking behaviour.[10,11] Smoking is associated with socio-economic,[12] social, and cultural factors which undergo changes following migration to new environments. In the Netherlands, researchers observed socio-economic gradients in smoking (smoking prevalence is higher as you have a lower socioeconomic group) among some ethnic minority populations, but not in sub-Saharan ethnic groups.[12] Inverse socio-economic gradients in smoking cessation (smoking cessation rate is lower as you have a lower socio-economic group) were recognised in other research.[13] Lower SES is associated with higher smoking possibly through mechanisms that include using smoking as a coping mechanism for stressful lives,[14,15] and less access to more costly and more effective options for smoking cessation.[16,17]

Few studies examined how much these differences in smoking and former smoking among ethnic minority groups relative to the Dutch were explained by socio-economic factors using both educational level and occupational level as socioeconomic indicators.[12,18]

In addition to ethnic differences in smoking, there are gender differences in tobacco usage, smoking behaviour,[19] acceptability of smoking, self-reporting of smoking, smoking cessation and factors that may influence the continuation of smoking globally.[20] It is well documented that men have higher smoking rates and higher daily cigarette consumption compared to women but this gender gap may vary among different ethnic groups and populations. We aimed to assess ethnic differences in smoking prevalence and former smoking among the Dutch, Ghanaian, Moroccan, Turkish, African-Surinamese and South-Asian Surinamese origin ethnic groups in the Netherlands, and to study to what extent these ethnic differences could be possibly



For peer review only - http://bmjopen.bmj.com/site/about/guidelines.xhtml

explained by differences in SES. In order to be able to address this general aim, we checked whether smoking prevalence and former smoking was related to low SES not only among those of Dutch origin, but also among the ethnic minority groups. We also calculated age-standardized prevalences of current smoking and former smoking by ethnic group and gender, and compared differences in smoking prevalence and former smoking in each minority ethnic group relative to the Dutch host population.

## METHODS

For peer review only

### Study design and setting

Baseline data from the multi-ethnic HELIUS (**H**ealthy **LI**ife in an **U**rban **S**etting) study were used. Ethical approval was granted by the Institutional Review Board of the Academic Medical Center at the University of Amsterdam, and all participants provided informed consent. In brief, HELIUS is investigating the patterns of health and health care utilization among the largest ethnic groups resident in Amsterdam, the Netherlands, including the Dutch host population and the ethnic minority groups of Ghanaian, Moroccan, Turkish, South-Asian Surinamese and African Surinamese origin.[1]

### Participants

Participants aged 18 to 70 years were randomly sampled from the Amsterdam municipal population register, stratified by ethnicity. Participants' ethnicity was defined according to the country of birth of the participant as well as that of his/her parents, which is currently the most widely accepted and most valid assessment of ethnicity in the Netherlands.[21] Specifically, a participant was considered as of ethnic minority origin if he/she fulfilled either of the following criteria: 1) he/she was born abroad and has at least one parent born abroad (first generation); or 2) he/she was born in the Netherlands but both his/her parents were born abroad (second generation). The Surinamese group was further classified according to self-reported ethnic origin into 'African', 'South-Asian', or other. Participants were considered of European Dutch ethnic origin (henceforth, Dutch) if the participant and both parents were born in the Netherlands. Data for the HELIUS study were collected via the use of a questionnaire and a physical examination from January 2011 to November 2015. For the current study, we used data of 23,942 participants for whom questionnaire data were available. Participants with an unknown/other ethnicity (n=50), Javanese Surinamese (n=250) or other/unknown



For peer review only - http://bmjopen.bmj.com/site/about/guidelines.xhtml

Surinamese ethnicity (n=286), and those with missing data on smoking status (n=107), marital status (n=134), and/or educational level (n=186) were excluded, resulting in a total sample size of 22,929 participants which was used in the current analyses.

**Smoking variables**

Smoking status was determined by the question 'Do you smoke at all?' Current smoking prevalence was calculated as the percentage of individuals in each ethnic group who responded 'yes' to this question. The prevalence of former smoking, also known as quit ratio percent,[22] in each ethnic group was determined by dividing the number of ex-smokers, (those who responded 'No, but I used to smoke') by the number of ever-smokers (the sum of ex and current smokers).

**Socioeconomic status variables**

The highest level of educational qualification attained (either in the Netherlands or in the country of origin) was grouped into three categories: 1) never been to school, or had elementary schooling only, or lower vocational schooling, or lower secondary schooling; 2) intermediate vocational schooling or higher secondary education schooling and; 3) higher vocational schooling or university level education. Occupational level was classified according to Dutch Standard Occupational Classification system for 2010.[23] This document provides an extensive systematic list of all professions in the Dutch system. Based on this document, occupational level was classified into 'elementary', 'lower', 'intermediate', 'higher', or 'graduate', based on job title and job description, including a question on fulfilling an executive function. Occupation-related SES was created by combining employment status (unemployed, not in working force (retired, student or full-time homeworker), incapacitated, or employed) with occupational level (elementary, lower, middle, higher or graduate level) among those who were employed. This resulted in the following categories: 1) unemployed, 2) not in the workforce, 3) incapacitated 4) employed at elementary/lower occupational level, and 5) employed at intermediate to graduate occupational level. The sixth category represented participants who were employed but there was insufficient data to define their occupational level.

**Statistical methods**



For peer review only - http://bmjopen.bmj.com/site/about/guidelines.xhtml

Men and women were analysed separately since literature shows that smoking behaviour, including the usage and reasons for using tobacco and cessation, differs substantially by gender.[24] To make fair comparisons of the smoking prevalence across all ethnic groups, age-standardized prevalence rates for current smoking and former smoking were calculated using the entire HELIUS population as the standard population, stratified by 10-year age groups. A likelihood ratio test was used to test whether there were interactions between ethnicity and educational level and between ethnicity and occupation-related SES.

A Poisson regression model with a robust variance estimator was used to directly estimate adjusted prevalence ratios with 95% confidence intervals for current smoking and former smoking in each ethnic minority group relative to the Dutch.[25] Prevalence ratios have been widely recommended in the literature as the preferred estimate if the outcome of interest is common as odds ratio tends to greatly overestimate the risk ratio in cross-sectional studies.[25,26]

The SES of ethnic minority groups living in high-income countries may be lower than the native host population and the relationship between SES and risk factors for health is skewed in that lower SES groups have increased risk factor tendencies and worse health outcomes than the more economically advantaged groups.[27] Since we anticipated that SES may lie on the causal pathway between ethnicity and smoking behaviour,[1] we expect that the relationship between ethnicity and smoking will be mediated by SES. Therefore, we assessed the extent to which socio-economic factors explained ethnic variations in current smoking and former smoking by adjusting ethnic differences in smoking and former smoking for educational level and occupation-related SES along with age and marital status. This was compared to the models adjusted for age and marital status only.

The presence of significant interactions between ethnicity and educational level ($p<0.0001$) and between ethnicity and occupation-related SES ($p<0.0001$) in both genders overall were observed. We therefore further stratified the analysis by ethnic group and gender to assess the relationship between SES and smoking prevalence and former smoking while adjusting for age and marital status. All analyses were performed using STATA Version 14.1 and bar charts were plotted using Excel 2013.

## RESULTS



For peer review only - http://bmjopen.bmj.com/site/about/guidelines.xhtml

For peer review only

**Socio-demographic characteristics of study population**

Table 1 shows the characteristics of the study population by gender and ethnic groups. On average, Moroccan and Turkish participants were younger than the Dutch, Ghanaian and Surinamese groups. Dutch men and women were more highly educated and had the lowest proportion of unemployed participants as compared with the ethnic minority groups.

**Current smoking**

Among men, smoking was more common in those of Turkish, African Surinamese and South-Asian Surinamese origin than Moroccan and Dutch origin while Ghanaians smoked the least (Figure 1). Among women, fewer Ghanaian and Moroccan women smoked compared to Dutch, Turkish, African Surinamese and South-Asian Surinamese women (Figure 1). After adjustment for age and marital status, men of Turkish, African Surinamese and South-Asian Surinamese origin were significantly more likely than men of Dutch origin to be current smokers, whereas Moroccan men did not significantly differ from the Dutch (Table 2, model 1). Additional adjustment for SES variables reduced the increased prevalence ratio of current smoking observed among Turkish, South-Asian Surinamese and African-Surinamese men relative to Dutch men, however, they still smoked significantly more than the Dutch (Table 2, models 2). The direction of the association changed for Moroccan men from 10% more likely to 18% less likely to smoke than Dutch men and the likelihood that Ghanaian men were less likely to smoke increased from 74% to 81% less likely (95% CI 85% to 75%) than Dutch men to be smokers.

Among women, only Turkish women were significantly more likely to be current smokers as compared with Dutch women, whereas Moroccan, South-Asian Surinamese and Ghanaian women were significantly less likely to be current smokers than women of Dutch origin (Table 2, model 1). For women, after additional adjustment for SES, women from all ethnic minority groups except Turkish were significantly less likely to smoke compared with Dutch women. Turkish women did not significantly differ from Dutch women anymore, after adjustment for SES (Table 2, models 2), whereas adjustment for SES made African-Surinamese women significantly less likely to be smokers than Dutch women.



For peer review only - http://bmjopen.bmj.com/site/about/guidelines.xhtml

For peer review only

Table 1: Socio-economic characteristics of the study population by ethnicity and sex

| | Dutch | Moroccan | Turkish | African Surinamese | South-Asian Surinamese | Ghanaian |
|---|---|---|---|---|---|---|
| **TOTAL SAMPLE SIZE N=22929** | **N=4598** | **N=4262** | **N=3985** | **N=4369** | **N=3320** | **N=2395** |
| **MEN N=9745** | **N=2115** | **N=1601** | **N=1800** | **N=1764** | **N=1536** | **N=929** |
| **Age, mean (SD)** | 46.9 (13.8) | 41.5 (12.9) | 40.2 (12.4) | 47.5 (13.4) | 44.3 (13.8) | 46.2 (12.0) |
| **Education, n (%)** | | | | | | |
| Higher vocational/ university | 1257 (59.4) | 297 (18.6) | 278 (15.4) | 312 (17.7) | 360 (23.4) | 85 (9.2) |
| Intermediate vocational/ higher secondary | 497 (23.5) | 539 (33.7) | 527 (29.3) | 608 (34.5) | 484 (31.5) | 283 (30.5) |
| Lower vocational or secondary and below | 361 (17.1) | 765 (47.8) | 995 (55.3) | 844 (47.9) | 692 (45.1) | 561 (60.4) |
| **Occupation, n (%)** | | | | | | |
| Unemployed | 124 (5.9) | 271 (17.0) | 285 (16.1) | 323 (18.5) | 227 (15.0) | 158 (16.1) |
| Not in the working force$^{\pi}$ | 338 (16.0) | 127 (8.0) | 129 (7.3) | 195 (11.2) | 180 (11.9) | 66 (7.2) |
| Incapacitated$^{\beta}$ | 57 (2.7) | 166 (10.4) | 158 (8.9) | 132 (7.6) | 126 (8.3) | 51 (5.5) |
| Employed at elementary/lower occupational level | 186 (8.8) | 515 (32.3) | 681 (38.3) | 529 (30.4) | 389 (25.6) | 517 (56.1) |
| Employed at middle to graduate occupational level | 1370 (64.8) | 452 (28.4) | 462 (26.0) | 510 (29.3) | 555 (36.6) | 94 (10.2) |
| Employed but unknown occupational level* | 38 (1.8) | 63 (4.0) | 61 (3.4) | 54 (3.1) | 41 (2.7) | 35 (3.8) |
| **WOMEN N=13184** | **N=2483** | **N=2661** | **N=2185** | **N=2605** | **N=1784** | **N=1466** |
| **Age, mean (SD)** | 45.4 (14.2) | 38.6 (13.0) | 39.5 (12.4) | 47.5 (12.4) | 45.7 (13.3) | 42.9 (11.0) |
| **Education, n (%)** | | | | | | |
| Higher vocational/ university | 1522 (61.3) | 439 (16.5) | 304 (13.9) | 672 (25.8) | 390 (21.9) | 66 (4.5) |
| Intermediate vocational/ higher secondary | 516 (20.8) | 633 (29.0) | 923 (34.7) | 958 (36.8) | 513 (28.8) | 333 (22.7) |
| Lower vocational or secondary and below | 445 (17.9) | 1299 (48.8) | 1248 (57.1) | 975 (37.4) | 881 (49.4) | 1067 (72.8) |
| **Occupation, n (%)** | | | | | | |
| Unemployed | 130 (5.3) | 389 (14.8) | 300 (13.9) | 385 (14.9) | 277 (15.7) | 408 (28.4) |
| Not in the working force$^{\pi}$ | 465 (18.8) | 1048 (39.9) | 792 (36.8) | 323 (12.5) | 297 (16.8) | 115 (8.0) |
| Incapacitated$^{\beta}$ | 84 (3.4) | 177 (6.7) | 199 (9.2) | 267 (10.3) | 179 (10.1) | 161 (11.2) |
| Employed at elementary/lower occupational level | 240 (9.7) | 349 (13.3) | 380 (17.6) | 417 (16.1) | 371 (21.0) | 611 (42.6) |
| Employed at middle to graduate occupational level | 1532 (61.9) | 607 (23.1) | 615 (34.8) | 1121 (43.4) | 615 (34.8) | 104 (7.3) |
| Employed but unknown occupational level* | 25 (1.0) | 59 (2.2) | 31 (1.8) | 73 (2.8) | 31 (1.8) | 36 (2.5) |

*working but did not provide sufficient information when describing their jobs; $^{\pi}$ retired or student or full-time homeworker; $^{\beta}$ disabled or unfit for work



For peer review only - http://bmjopen.bmj.com/site/about/guidelines.xhtml

Table 2: Prevalence and Adjusted Prevalence Ratios (PRs) with 95% CIs of current smoking and former smoking of ethnic minority groups compared with Dutch

| | Current smoking | | | Former smoking/ Quit ratio percent | | |
|---|---|---|---|---|---|---|
| Ethnic group | | Model 1* | Model 2** | | Model 1* | Model 2** |
| | Crude Prevalence n/N (%) | PR (95% CI) | PR (95% CI) | Crude Prevalence n/N (%) | PR (95% CI) | PR (95% CI) |
| **Men** | | | | | | |
| **Dutch** | 562/2115 (26.6) | 1.00 | 1.00 | 827/1389 (59.5) | 1.00 | 1.00 |
| Turkish men | 772/1800 (42.9) | 1.69 (1.54, 1.86) | 1.23 (1.11, 1.36) | 442/1214 (36.4) | 0.67 (0.62, 0.74) | 0.79 (0.72, 0.87) |
| African Surinamese | 780/1764 (44.2) | 1.55 (1.41, 1.69) | 1.20 (1.09, 1.32) | 363/1143 (31.8) | 0.57 (0.52, 0.63) | 0.64 (0.58, 0.71) |
| South-Asian Surinamese | 645/1536 (42.0) | 1.53 (1.40, 1.68) | 1.21 (1.10, 1.33) | 239/884 (27.0) | 0.50 (0.45, 0.56) | 0.57 (0.50, 0.64) |
| Moroccan | 446/1601 (27.9) | 1.10 (0.99, 1.23) | 0.82 (0.73, 0.92) | 432/878 (49.2) | 0.88 (0.82, 0.95) | 1.02 (0.94, 1.12) |
| Ghanaian | 69/929 (7.4) | 0.26 (0.20, 0.33) | 0.19 (0.15, 0.24) | 118/187 (63.1) | 1.08 (0.96, 1.22) | 1.28 (1.12, 1.45) |
| **Women** | | | | | | |
| **Dutch** | 593/2483 (23.9) | 1.00 | 1.00 | 918/1511 (60.8) | 1.00 | 1.00 |
| Turkish women | 647/2185 (29.6) | 1.26 (1.14, 1.40) | 1.03 (0.92, 1.15) | 253/900 (28.1) | 0.54 (0.48, 0.61) | 0.66 (0.59, 0.75) |
| African Surinamese | 660/2605 (25.3) | 0.95 (0.86, 1.05) | 0.75 (0.68, 0.83) | 470/1130 (41.6) | 0.75 (0.69, 0.81) | 0.85 (0.78, 0.92) |
| South-Asian Surinamese | 370/1784 (20.7) | 0.82 (0.73, 0.92) | 0.65 (0.58, 0.73) | 193/563 (34.3) | 0.64 (0.56, 0.72) | 0.73 (0.65, 0.83) |
| Moroccan | 145/2661 (5.5) | 0.23 (0.19, 0.27) | 0.19 (0.16, 0.23) | 93/238 (39.1) | 0.87 (0.74, 1.03) | 1.01 (0.86, 1.19) |
| Ghanaian | 37/1466 (2.5) | 0.09 (0.06, 0.12) | 0.06 (0.05, 0.09) | 75/112 (67.0) | 1.24 (1.07, 1.43) | 1.57 (1.34, 1.83) |

*Model 1: Adjusted for age and marital status; **Model 2: Adjusted for age, marital status, education and occupation-related SES

For peer review only

**Former smoking**

Among both sexes, the highest prevalence of former smoking was in Ghanaians while the lowest was in African-Surinamese men and Turkish women (Figure 2). All ethnic minority groups were significantly less likely to quit smoking as compared with Dutch, except for Ghanaians who were more likely to have quit (although not statistically significant in men) (Table 2, models 1). For former smoking, although the Turkish, Surinamese and Moroccan ethnic groups appeared to be less likely to quit smoking than the Dutch, the lower likelihoods were partly explained by socio-economic factors (Table 2, model 2). Ghanaians were even more likely to have a higher former smoking rate than the Dutch after socio-economic factors were accounted for.

**The relationship between SES and smoking prevalence and former smoking within each ethnic group**

Within each ethnic group, we observed SES inequalities in smoking prevalence (Table 3). For both sexes, participants with higher educational levels were less likely to smoke than those with lower educational levels in all ethnic groups, although not always statistically significant. Although very wide confidence intervals were obtained for Ghanaian women, those with higher vocational schooling or university level education smoked more than women with little or no education.

Compared to those employed in middle to graduate level positions, all unemployed men smoked more in all ethnic groups.  Although not always statistically significant all unemployed and lower level professional women except Ghanaians were more likely to smoke. Persons who were disabled or unfit for work (incapacitated) also had a higher prevalence of smoking in all ethnic groups, although not statistically significant.

Within each ethnic group, SES inequalities were observed in former smoking (Table 4). For both sexes, participants with higher levels of education were more likely to quit smoking although not significant for Moroccan men and women. Higher educated Ghanaian women seemed less likely to quit smoking yet this was not significant. For both sexes, unemployed participants were less likely to quit compared to the middle or graduate level. Participants employed in lower level professional jobs were less likely to quit than those employed at the middle or graduate level except for Ghanaian women who appeared to be more likely to quit. A similar lower likelihood of quitting was observed among the incapacitated with the exception of Moroccan women.



For peer review only - http://bmjopen.bmj.com/site/about/guidelines.xhtml

**Table 3: Current smoking prevalence ratios (95% CI) among men and women of different educational levels and occupation-related socio-economic status within each ethnic group**

| Indicator of socioeconomic status | Dutch | South-Asian Surinamese | African Surinamese | Turkish | Moroccan | Ghanaian |
|---|---|---|---|---|---|---|
| | PR (95% CI)* | PR (95% CI)* | PR (95% CI)* | PR (95% CI)* | PR (95% CI)* | PR (95% CI)* |
| **Men** | | | | | | |
| **Education** | | | | | | |
| Higher vocational school/ university | 0.57 (0.47, 0.69) | 0.44 (0.36, 0.53) | 0.65 (0.55, 0.78) | 0.50 (0.41, 0.62) | 0.62 (0.49, 0.80) | 0.28 (0.07, 1.19) |
| Intermediate vocational/ higher secondary | 0.57 (0.57, 0.87) | 0.69 (0.61, 0.80) | 0.92 (0.82, 1.03) | 0.83 (0.73, 0.94) | 0.83 (0.70, 0.99) | 0.78 (0.46, 1.33) |
| Lower vocational and below | 1.00 | 1.00 | 1.00 | 1.00 | 1.00 | 1.00 |
| **Occupation-related SES** | | | | | | |
| Unemployed | 1.91 (1.53, 2.38) | 1.69 (1.43, 1.99) | 1.58 (1.35, 1.85) | 1.32 (1.11, 1.56) | 1.81 (1.46, 2.26) | 5.10 (1.17, 22.30) |
| Not in the working force$^{\pi}$ | 1.11 (0.87, 1.42) | 0.99 (0.76, 1.29) | 1.22 (0.96, 1.54) | 0.73 (0.53, 1.02) | 0.60 (0.36, 1.01) | 2.37 (0.37, 15.33) |
| Incapacitated$^{\beta}$ | 1.58 (1.10, 2.30) | 1.30 (1.02, 1.65) | 1.48 (1.21, 1.81) | 1.11 (0.88, 1.41) | 1.49 (1.11, 2.00) | 4.34 (0.83, 22.77) |
| Employed at elementary/lower occupational level | 1.50 (1.20, 1.87) | 1.57 (1.34, 1.84) | 1.32 (1.13, 1.53) | 1.27 (1.10, 1.46) | 1.33 (1.07, 1.64) | 3.37 (0.79, 14.37) |
| Employed at middle to graduate occupational level | 1.00 | 1.00 | 1.00 | 1.00 | 1.00 | 1.00 |
| **Women** | | | | | | |
| **Education** | | | | | | |
| Higher vocational school/university | 0.39 (0.33, 0.46) | 0.61 (0.47, 0.80) | 0.58 (0.48, 0.70) | 0.72 (0.56, 0.91) | 0.87 (0.53, 1.45) | 6.02 (2.23, 16.25) |
| Intermediate vocational/ higher secondary | 0.60 (0.50, 0.73) | 0.87 (0.70, 1.08) | 0.75 (0.65, 0.87) | 1.01 (0.87, 1.17) | 0.90 (0.59, 1.37) | 0.56 (0.19, 1.62) |
| Lower vocational and below | 1.00 | 1.00 | 1.00 | 1.00 | 1.00 | 1.00 |
| **Occupation-related SES** | | | | | | |
| Unemployed | 1.27 (0.96, 1.67) | 1.66 (1.30, 2.12) | 1.54 (1.29, 1.84) | 1.16 (0.94, 1.43) | 1.42 (0.90, 2.22) | 0.70 (0.18, 2.68) |
| Not in the working force$^{\pi}$ | 1.09 (0.86, 1.38) | 1.04 (0.74, 1.45) | 1.11 (0.86, 1.45) | 1.03 (0.85, 1.25) | 0.86 (0.53, 1.39) | 1.44 (0.33, 6.25) |
| Incapacitated$^{\beta}$ | 1.38 (1.01, 1.90) | 1.82 (1.34, 2.47) | 1.47 (1.17, 1.84) | 1.22 (0.95, 1.56) | 1.40 (0.77, 2.57) | 0.76 (0.17, 3.49) |
| Employed at elementary/lower occupational level | 1.69 (1.39, 2.07) | 1.23 (0.94, 1.61) | 1.34 (1.11, 1.62) | 1.29 (1.06, 1.57) | 1.22 (0.75, 1.99) | 0.44 (0.12, 1.67) |
| Employed at middle to graduate occupational level | 1.00 | 1.00 | 1.00 | 1.00 | 1.00 | 1.00 |

-*PR Adjusted for age and marital status; $^{\pi}$ retired or student or full-time homeworker; $^{\beta}$ disabled or unfit for work



For peer review only

For peer review only - http://bmjopen.bmj.com/site/about/guidelines.xhtml

Table 4: Former smoking prevalence ratios (95% CI) among men and women of different educational levels and occupation-related socio-economic status within each ethnic group

| Indicator of socioeconomic status | Dutch | South-Asian Surinamese | African Surinamese | Turkish | Moroccan | Ghanaian |
|---|---|---|---|---|---|---|
| | PR (95% CI)* | PR (95% CI)* | PR (95% CI)* | PR (95% CI)* | PR (95% CI)* | PR (95% CI)* |
| **Men** | | | | | | |
| **Education** | | | | | | |
| Higher vocational school/ university | 1.28 (1.14, 1.45) | 1.84 (1.44, 2.37) | 1.38 (1.12, 1.69) | 1.54 (1.27, 1.87) | 1.16 (0.95, 1.41) | 1.38 (1.05, 1.82) |
| Intermediate vocational/ higher secondary | 1.19 (1.04, 1.37) | 1.09 (0.82, 1.44) | 0.97 (0.80, 1.19) | 1.12 (0.94, 1.34) | 1.02 (0.88, 1.19) | 1.09 (0.84, 1.41) |
| Lower vocational and below | 1.00 | 1.00 | 1.00 | 1.00 | 1.00 | 1.00 |
| **Occupation-related SES** | | | | | | |
| Unemployed | 0.71 (0.56, 0.89) | 0.59 (0.41, 0.86) | 0.58 (0.44, 0.76) | 0.85 (0.67, 1.08) | 0.77 (0.61, 0.96) | 0.66 (0.47, 0.91) |
| Not in the working force$^{\pi}$ | 1.02 (0.91, 1.14) | 0.91 (0.64, 1.31) | 0.81 (0.62, 1.07) | 0.89 (0.60, 1.32) | 0.99 (0.75, 1.29) | 0.65 (0.36, 1.15) |
| Incapacitated$^{\beta}$ | 0.83 (0.63, 1.09) | 0.79 (0.52, 1.17) | 0.68 (0.48, 0.97) | 0.96 (0.74, 1.23) | 0.88 (0.70, 1.09) | 0.77 (0.51, 1.18) |
| Employed at elementary/lower occupational level | 0.82 (0.69, 0.98) | 0.57 (0.43, 0.76) | 0.72 (0.58, 0.89) | 0.85 (0.70, 1.03) | 0.93 (0.78, 1.11) | 0.63 (0.47, 0.84) |
| Employed at middle to graduate occupational level | 1.00 | 1.00 | 1.00 | 1.00 | 1.00 | 1.00 |
| **Women** | | | | | | |
| **Education** | | | | | | |
| Higher vocational school/university | 1.71 (1.52, 1.93) | 1.99 (1.33, 2.31) | 1.31 (1.10, 1.55) | 1.73 (1.33, 2.25) | 1.09 (0.71, 1.66) | 0.72 (0.36, 1.41) |
| Intermediate vocational/ higher secondary | 1.41 (1.21, 1.62) | 1.75 (1.33, 2.31) | 1.18 (1.00, 1.38) | 1.21 (0.95, 1.56) | 1.12 (0.78, 1.61) | 1.28 (0.98, 1.65) |
| Lower vocational and below | 1.00 | 1.00 | 1.00 | 1.00 | 1.00 | 1.00 |
| **Occupation-related SES** | | | | | | |
| Unemployed | 0.89 (0.73, 1.08) | 0.62 (0.44, 0.88) | 0.73 (0.58, 0.92) | 0.66 (0.48, 0.91) | 0.97 (0.61, 1.53) | 0.95 (0.60, 1.51) |
| Not in the working force$^{\pi}$ | 0.92 (0.83, 1.03) | 0.77 (0.55, 1.07) | 0.98 (0.79, 1.20) | 0.54 (0.40, 0.73) | 1.02 (0.64, 1.62) | 0.52 (0.22, 1.22) |
| Incapacitated$^{\beta}$ | 0.86 (0.69, 1.08) | 0.57 (0.38, 0.87) | 0.85 (0.69, 1.05) | 0.65 (0.45, 0.96) | 1.20 (0.75, 1.91) | 0.97 (0.55, 1.71) |
| Employed at elementary/lower occupational level | 0.76 (0.65, 0.89) | 0.48 (0.32, 0.73) | 0.76 (0.61, 0.94) | 0.60 (0.43, 0.82) | 0.84 (0.49, 1.44) | 1.07 (0.69, 1.66) |
| Employed at middle to graduate occupational level | 1.00 | 1.00 | 1.00 | 1.00 | 1.00 | 1.00 |

-*PR Adjusted for age and marital status; $^{\pi}$ retired or student or full-time homeworker; $^{\beta}$ disabled or unfit for work



For peer review only - http://bmjopen.bmj.com/site/about/guidelines.xhtml

## DISCUSSION

### Key findings

The prevalence of current smoking and former smoking of adult men and women of minority ethnic groups living in Amsterdam is different from the Dutch host population. Accounting for socio-economic factors, the analysis showed that Turkish, South-Asian Surinamese and African Surinamese men smoked more than Dutch men while Ghanaian and Moroccan men smoked less. The smoking prevalence of Turkish women was similar to Dutch women while Ghanaian, Moroccan, South-Asian Surinamese and African Surinamese women smoked less. Only the Ghanaian ethnic group was more likely to quit than the Dutch. Socio-economic factors influenced current smoking and former smoking prevalence, but more so among men than among women and differently depending on the ethnic minority group. SES contributed to the higher prevalence of smoking among Turkish, Surinamese and Moroccan men and Turkish women, but diminished the estimates for Surinamese, Moroccan and Ghanaian women who were less likely to smoke than the Dutch. Socio-economic factors also accounted for part of the lower prevalence of former smoking observed among the ethnic minority groups relative to the Dutch.

### Discussion of key findings

Smoking behaviour differed among the various ethnic minority groups compared with the Dutch. The prevalence of smoking among Ghanaians in the Netherlands has not been previously studied. Comparable studies in Amsterdam, which used the same country of birth criteria for ethnicity, indicates that smoking prevalence among adults aged 35-60 years has reduced over the last decade (from 2000/2003 to 2011/2015) in Moroccan, Turkish, and Surinamese groups.[12] Age-standardized estimates of smoking prevalence among Turkish men (63%) declined to ~41.7%, and from 55% to 44.8% in Surinamese men, but remained fairly constant in Moroccan men (~29%). However, a slight increase in smoking prevalence from <1% to ~2.6% among Moroccan women was observed. The presence of stronger tobacco control policies in the Netherlands after 2001 may be a contributing factor for the decline in smoking prevalence among Turkish and Surinamese men over the last decade bringing it closer to Dutch men.[28]

When referring to estimates of smoking from population-based studies conducted in the respective countries of origin, we observed more variability between women in the home country vs women in Amsterdam: 13% vs



For peer review only - http://bmjopen.bmj.com/site/about/guidelines.xhtml

27% (Turkish),[29] 9.9% vs 24.2% (Surinamese),[30] 3.3% vs 4.3% (Moroccan),[31] and 0.3% vs 2.9% (Ghanaian).[32] Whereas for men, closer parallels between men in their home countries and men in Amsterdam: 41% vs 40.5% (Turkish),[29] 38.4% vs 44.8% (Surinamese),[30] ; 31.5% vs 26.8% (Moroccan),[31]; and 8.9% vs 7.5% (Ghanaian) were detected.[32] These differences, especially among women, allude to the possible impact of acculturation, and different social norms on smoking.[9-11,33]

From our findings, lower SES was related to higher smoking prevalence and lower former smoking more in men in most ethnic groups and is consistent with findings from past research.[34-36] Smoking was more common among lower educated men of Turkish and host populations in a study conducted in Germany and the Netherlands.[37] However, higher educated Turkish women smoked more than lower educated while the reverse was evident in the host population.[37] Higher smoking among the more educated women was observed only in Ghanaian women but very wide confidence intervals were obtained due to small numbers. Higher smoking among the more educated women in Amsterdam may flag the signs of the early stages of tobacco smoking epidemic among Ghanaian women,[38,39] who may be challenging cultural norms and taboos towards smoking present especially among women in the Ghanaian community.

For former smoking, higher prevalences among the Dutch than African and South-Asian Surinamese are known.[18] This study confirmed that Turkish and Surinamese smokers have lower former smoking prevalence while Ghanaian smokers are more likely to quit than the Dutch. There were indications that Moroccan and Dutch had similar quit ratios. Others factors including lack of social support, more stressful lives, or living in deprived neighbourhoods may be ways in which lower SES indirectly influences smoking cessation.[40]

Higher rates of smoking were observed among both men and women employed in lower level professional jobs than those in higher professional jobs of all ethnic groups except Ghanaian and Moroccan men in which the difference may not have been detected due to small numbers. Enforcing restrictions on smoking on the workplace can make it more difficult for even workers in lower level jobs to smoke on the job.[41] This emergence of socioeconomic gradients in smoking among ethnic groups reflects on the recent findings in the Netherlands which shows increasing hazard ratios for acute myocardial infarction and stroke across medium and lower SES ethnic groups compared to the higher SES groups.[42,43] Since smoking is attributable to death from a number of causes including myocardial infarction and stroke, reducing smoking and or increasing



For peer review only - http://bmjopen.bmj.com/site/about/guidelines.xhtml

cessation among ethnic minority groups can help reduce the high all-cause mortality and cardiovascular disease incidence already seen among Turkish and Surinamese in the Netherlands.[2,44,45]

The strengths of the HELIUS study is that it utilized large sample sizes in each ethnic group and participants were recruited in a systematic manner to reflect a representative sample of adults in each ethnic group living in Amsterdam.[1] Smoking status may be underreported since biochemical methods were not used to verify self-reported smoking statuses. People tend to underreport undesirable health behaviours especially those stigmatized within the wider society.[46] Urine analysis of cotinine levels, the main metabolite of nicotine, is a useful biochemical indicator that can be used to validate current smoking among participants.[47] This study was therefore limited in its ability to accurately confirm current smoking, and thus it may be underestimated in this study. Our research did not focus on analysing what current smokers smoked, however the majority used cigarettes rather than cigars and packages of pipe tobacco. Smokers were not asked about the use of other tobacco products. It might be that some ethnic groups may have a cultural preference to use other smokeless forms of tobacco or other products containing sources of tobacco, so the use of tobacco might have not been fully captured in this study. Smoking cessation was assessed by using quit ratios, which is a cumulative measure of a number of former smokers in the ethnic group, which occurred over time and not necessarily an indicator of a recent smoking cessation or assessment at the individual level.[41]

## CONCLUSION

Current smoking and former smoking varies by ethnic group in the Netherlands. The higher levels of smoking prevalence and the lower levels of former smoking among some ethnic minority groups compared to the Dutch are largely, but not completely, explained by socioeconomic factors. Differences in SES are related to smoking prevalence and cessation among ethnic groups. Our findings imply that we would be better able to reduce smoking in the Netherlands by factoring in ethnic diversity in smoking that we now better understand. Anti-smoking policies designed to target different ethnic groups and those of lower socio-economic status may substantially reduce smoking particularly among men in the Netherlands. However, among Ghanaian women, smoking prevention measures should particularly include higher educated women.

For peer review only

## FUNDING

The HELIUS study is funded by the Dutch Heart Foundation (Hartstichting) (grant number 2010T084), the Netherlands Organization for Health Research and Development (ZonMw grant number 200500003), and the European Commission Seventh Framework Programme (FP-7) (grant number 278901). The views expressed in the paper are those of the authors, the funders had no participation in the writing of this paper.

## DECLARATION OF INTETESTS

None declared.

## AUTHOR CONTRIBUTIONS

The study idea was conceived my RB in collaboration with LS, JA,  and CA. The first draft of the paper was written by RB. LS, JA, CA, EMD, AEK, RP, MBS, all wrote and contributed to several updated versions.

## ACKNOWLEDGEMENTS

The HELIUS study is conducted by the Academic Medical Center Amsterdam and the Public Health Service of Amsterdam. Both organisations provided core support for HELIUS. We are most grateful to the participants of the HELIUS study and the management team, research nurses, interviewers, research assistants and other staff who have taken part in gathering the data of this study.

**Figure Legends**

Figure 1 Age-standardized prevalence of current smoking by gender and ethnicity

Figure 2 Age-standardized prevalence of former smoking by gender and ethnicity



For peer review only - http://bmjopen.bmj.com/site/about/guidelines.xhtml

For peer review only

## REFERENCES


1. Stronks K, Snijder M, Peters R, et al. Unravelling the impact of ethnicity on health in Europe: the HELIUS study. *BMC Public Health.* 2013;13(1):1-10 10.1186/1471-2458-13-402.
2. Bos V, Kunst AE, Keij-Deerenberg IM, Garssen J, Mackenbach JP. Ethnic inequalities in age- and cause-specific mortality in the Netherlands. *Int. J. Epidemiol.* 2004;33(5):1112-1119 10.1093/ije/dyh189.
3. World Health Organization. *WHO REPORT ON THE GLOBAL TOBACCO EPIDEMIC 2008.* Geneva: World Health Organization;2008
4. ASH, Health Development Agency. Smoking and Health Inequalities. London.
5. Centers for Disease Control and Prevention. Cigarette smoking among adults-United States 1998. *Morbidity and Mortality Weekly Report.* 2000;49(39):881-884
6. Department of Health. Smoking kills A white paper on tobacco. London: The Stationery Office; 1998.
7. Bos V. *Ethnic inequalities in mortality in the Netherlands and the role of socioeconomic status*. Rotterdam, the Netherlands: Department of Public Health, Erasmus MC Univeristy Medical Centre; 2005.
8. Scarborough P, Bhatnagar P, Kaur A, et al. *Ethnic Differences in Cardiovascular Disease 2010 Edition.* British Heart Foundation Health Promotion Research Group,Department of Public Health, University of Oxford;2010
9. Wilkinson AV, Spitz MR, Strom SS, et al. Effects of nativity, age at migration, and acculturation on smoking among adult Houston residents of Mexican descent. *Am. J. Public Health.* 2005;95(6):1043-1049 10.2105/AJPH.2004.055319).
10. Bethel JW, Schenker MB. Acculturation and smoking patterns among Hispanics: A review. *Am. J. Prev. Med.* 2005;29(2):143-148 doi:10.1016/j.ampere.2005.04.014.
11. Choi S, Rankin S, Stewart A, Oka R. Effects of acculturation on Smoking Behavior in Asian Americans: A Meta-Analysis. *J. Cardiovasc. Nurs.* 2008;23(1):67-73 10.1097/1001.JCN.0000305057.0000396247.f0000305052 10.1097/01.JCN.0000305057.96247.f2.
12. Nierkens V, de Vries H, Stronks K. Smoking in immigrants: do socioeconomic gradients follow the pattern expected from the tobacco epidemic? *Tob. Control.* 2006;15(5):385-391 10.1136/tc.2006.014902.
13. Fernandez E, Schiaffino A, García M, Borràs JM. Widening social inequalities in smoking cessation in Spain, 1987–1997. *J. Epidemiol. Community Health.* 2001;55(10):729-730 10.1136/jech.55.10.729.
14. Lantz PM, House JS, Mero RP, Williams DR. Stress, life events, and socioeconomic disparities in health: results from the Americans' Changing Lives Study. *J. Health Soc. Behav.* 2005;46(3):274-288
15. Layte R, Whelan CT. Explaining Social Class Inequalities in Smoking: The Role of Education, Self-Efficacy, and Deprivation. *European Sociological Review.* 2009;25(4):399-410 10.1093/esr/jcn022.
16. Manley MW, Griffin T, Foldes SS, Link CC, Sechrist RA. The role of health plans in tobacco control. *Annu. Rev. Public Health.* 2003;24:247-266 10.1146/annurev.publhealth.24.100901.140838.
17. Bauer JE, Hyland A, Li Q, Steger C, Cummings KM. A longitudinal assessment of the impact of smoke-free worksite policies on tobacco use. *Am. J. Public Health.* 2005;95(6):1024-1029 10.2105/ajph.2004.048678.
18. Agyemang C, Stronks K, Tromp N, et al. A cross-national comparative study of smoking prevalence and cessation between English and Dutch South Asian and African origin




For peer review only - http://bmjopen.bmj.com/site/about/guidelines.xhtml

populations: the role of national context. *Nicotine Tob. Res.* 2010;12(6):557-566 10.1093/ntr/ntq044.
19. Bauer T, Gohlmann S, Sinning M. Gender differences in smoking behavior. *Health Econ.* 2007;16(9):895-909 10.1002/hec.1259.
20. The Tobacco Atlas, Fifth Edition. American Cancer Society, Inc; 2015. http://www.tobaccoatlas.org/.
21. Stronks K, Kulu-Glasgow I, Agyemang C. The utility of 'country of birth' for the classification of ethnic groups in health research: the Dutch experience. *Ethn. Health.* 2009;14(3):255-269 10.1080/13557850802509206.
22. Kulik MC, Glantz SA. The smoking population in the USA and EU is softening not hardening. *Tob. Control.* 2015 doi:10.1136/tobaccocontrol-2015-052329.
23. Central Bureau of Statistics. *Standard Occupational Classification 2010.* 2010
24. National Institute on Drug Abuse, National Institutes of Health, U.S. Department of Health and Human Services. *Tobacco/Nicotine.* 2016
25. Coutinho LM, Scazufca M, Menezes PR. Methods for estimating prevalence ratios in cross-sectional studies. *Rev. Saude Publica.* 2008;42(6):992-998 http://dx.doi.org/10.1590/S0034-89102008000600003
26. Barros A, Hirakata V. Alternatives for logistic regression in cross-sectional studies: an empirical comparison of models that directly estimate the prevalence ratio. *BMC Med. Res. Methodol.* 2003;3(1):1-13 10.1186/1471-2288-3-21.
27. Richard Wilkinson, Marmot M, eds. *Social determinants of health: the solid facts.* 2nd ed. Denmark: World Health Organisation; 2003.
28. Bosdriesz JR, Nagelhout GE, Stronks K, Willemsen MC, Kunst AE. The association between tobacco control policy and educational inequalities in smoking cessation in the Netherlands from 1988 through 2011. *Nicotine Tob. Res.* 2015 10.1093/ntr/ntv004.
29. Republic of Turkey Ministry of Health. *Global Adult Tobacco Survey Turkey 2012.* Ankara2014
30. World Health Organization. *WHO country report on the global tobacco epidemic, 2012 country profile Suriname.* 2013
31. Tachfouti N, El RK, Berraho M, et al. Knowledge and attitude about antismoking legislation in Morocco according to smoking status. *East. Mediterr. Health J.* 2011;17(4):297-302
32. Owusu-Dabo E, Lewis S, McNeill A, Gilmore A, Britton J. Smoking uptake and prevalence in Ghana. *Tob. Control.* 2009;18(5):365-370 10.1136/tc.2009.030635.
33. Perez-Stable EJ, Ramirez A, Villareal R, et al. Cigarette smoking behavior among US Latino men and women from different countries of origin. *Am. J. Public Health.* 2001;91(9):1424-1430
34. Cavelaars AEJM, Kunst AE, Geurts JJM, et al. *Educational differences in smoking: international comparison.* Vol 3202000.
35. Faggiano F, Versino E, Lemma P. Decennial trends of social differentials in smoking habits in Italy. *Cancer Causes Control.* 2001;12(7):665-671 10.1023/A:1011247024979.
36. Galobardes B, Costanza MC, Bernstein MS, Delhumeau C, Morabia A. Trends in risk factors for lifestyle-related diseases by socioeconomic position in Geneva, Switzerland, 1993–2000: Health inequalities persist. *Am. J. Public Health.* 2003;93(8):1302-1309
37. Reiss K, Sauzet O, Breckenkamp J, Spallek J, Razum O. How immigrants adapt their smoking behaviour: comparative analysis among Turkish immigrants in Germany and the Netherlands. *BMC Public Health.* 2014;14:844 10.1186/1471-2458-14-844.
38. Pampel FC. Age and education patterns of smoking among women in high-income nations. *Soc. Sci. Med.* 2003;57(8):1505-1514 http://dx.doi.org/10.1016/S0277-9536(02)00543-9.
39. Pampel FC. Cigarette diffusion and sex differences in smoking. *J. Health Soc. Behav.* 2001;42(4):388-404

For peer review only



For peer review only - http://bmjopen.bmj.com/site/about/guidelines.xhtml

40. Businelle MS, Kendzor DE, Reitzel LR, et al. Mechanisms linking socioeconomic status to smoking cessation: a structural equation modeling approach. *Health Psychol.* 2010;29(3):262-273 10.1037/a0019285.

41. Burns DM. Smoking Cessation: Recent inidcators of what's working at a population level. *Smoking and Tobacco Control Monograph No. 12.* Vol 12: National Cancer Institute; 2000.

42. Agyemang C, van Oeffelen AA, Norredam M, et al. Socioeconomic inequalities in stroke incidence among migrant groups: analysis of nationwide data. *Stroke; a journal of cerebral circulation.* 2014;45(8):2397-2403 10.1161/strokeaha.114.005505.

43. Agyemang C, van Oeffelen AA, Bots ML, Stronks K, Vaartjes I. Socioeconomic inequalities in acute myocardial infarction incidence in migrant groups: has the epidemic arrived? analysis of nation-wide data. *Heart.* 2014;100(3):239-246 10.1136/heartjnl-2013-304721.

44. Agyemang C, van Oeffelen AAM, Norredam M, et al. Ethnic disparities in ischemic stroke, intracerebral hemorrhage, and subarachnoid hemorrhage incidence in the Netherlands. *Stroke.* 2014;45(11):3236-3242 10.1161/strokeaha.114.006462.

45. van Oeffelen AAM, Vaartjes I, Stronks K, Bots ML, Agyemang C. Incidence of acute myocardial infarction in first and second generation minority groups: Does the second generation converge towards the majority population? *Int. J. Cardiol.* 2013;168(6):5422-5429 http://dx.doi.org/10.1016/j.ijcard.2013.08.046.

46. Elkind AK. The social definition of women's smoking behaviour. *Soc. Sci. Med.* 1985;20(12):1269-1278 10.1016/0277-9536(85)90380-6.

47. Benowitz NL, Jacob P. Metabolism of nicotine to cotinine studied by a dual stable isotope method. *Clin. Pharmacol. Ther.* 1994;56(5):483-493 10.1038/clpt.1994.169.

For peer review only



For peer review only - http://bmjopen.bmj.com/site/about/guidelines.xhtml

For peer review only



For peer review only - http://bmjopen.bmj.com/site/about/guidelines.xhtml

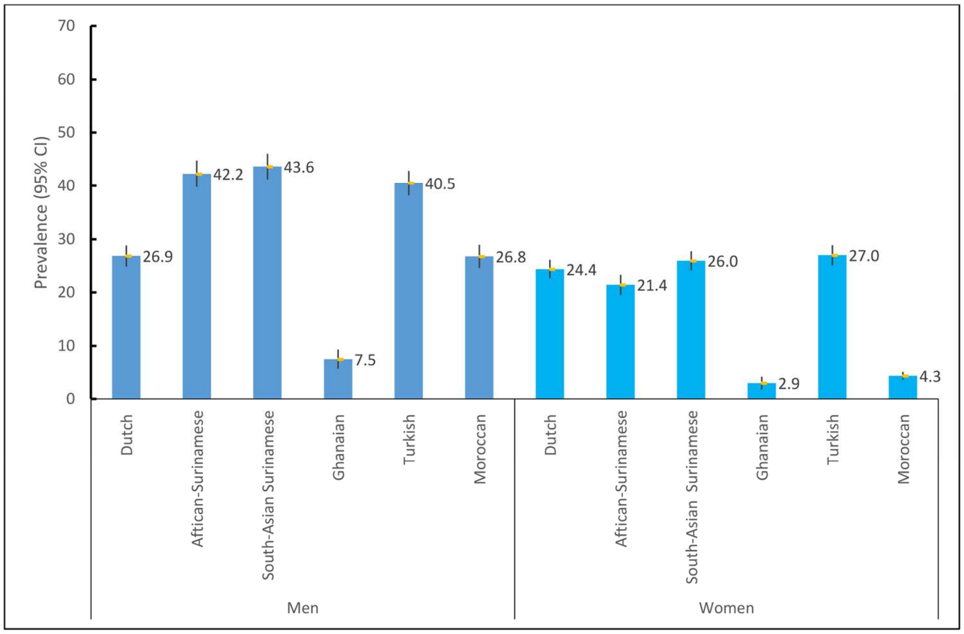


Figure 1 Age-standardized prevalence of current smoking by gender and ethnicity

165x107mm (300 x 300 DPI)

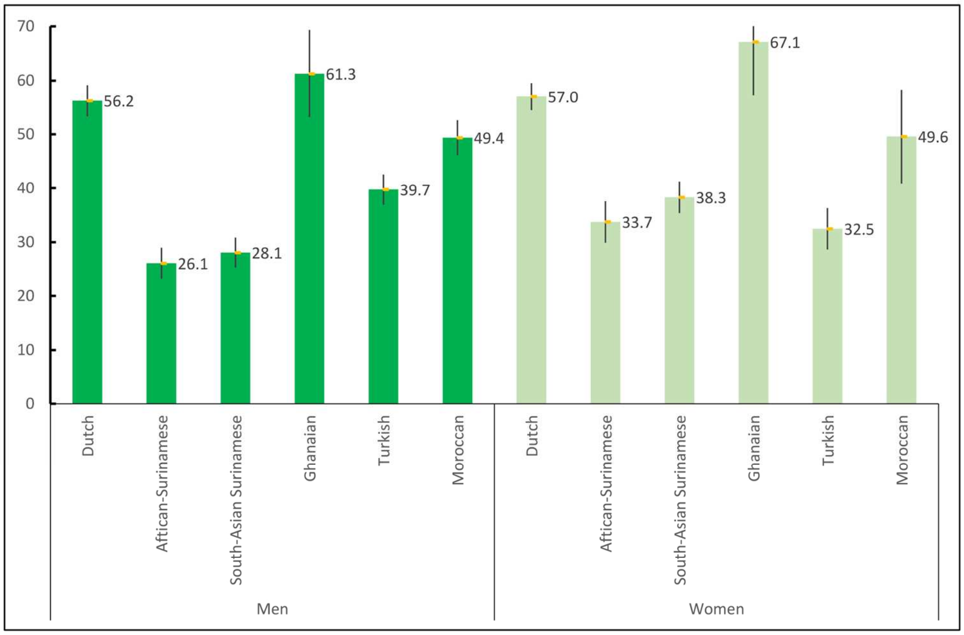


Figure 2 Age-standardized prevalence of former smoking by gender and ethnicity

165x107mm (300 x 300 DPI)

STROBE Statement—checklist of items that should be included in reports of observational studies

| | Item No | Recommendation | |
|---|---|---|---|
| **Title and abstract** | 1 | (*a*) Indicate the study's design with a commonly used term in the title or the abstract | ✓Abstract |
| | | (*b*) Provide in the abstract an informative and balanced summary of what was done and what was found | ✓ |
| **Introduction** | | | |
| Background/rationale | 2 | Explain the scientific background and rationale for the investigation being reported | ✓ page 5 |
| Objectives | 3 | State specific objectives, including any prespecified hypotheses | ✓ pages 5,6 |
| **Methods** | | | |
| Study design | 4 | Present key elements of study design early in the paper | ✓ page 6 |
| Setting | 5 | Describe the setting, locations, and relevant dates, including periods of recruitment, exposure, follow-up, and data collection | ✓ page 6 |
| Participants | 6 | (*a*) *Cohort study*—Give the eligibility criteria, and the sources and methods of selection of participants. Describe methods of follow-up<br>*Case-control study*—Give the eligibility criteria, and the sources and methods of case ascertainment and control selection. Give the rationale for the choice of cases and controls<br>*Cross-sectional study*—Give the eligibility criteria, and the sources and methods of selection of participants | ✓ pages 6,7 |
| | | (*b*) *Cohort study*—For matched studies, give matching criteria and number of exposed and unexposed<br>*Case-control study*—For matched studies, give matching criteria and the number of controls per case | |
| Variables | 7 | Clearly define all outcomes, exposures, predictors, potential confounders, and effect modifiers. Give diagnostic criteria, if applicable | ✓ page 5 and 6 |
| Data sources/ measurement | 8* | For each variable of interest, give sources of data and details of methods of assessment (measurement). Describe comparability of assessment methods if there is more than one group | ✓ page 7 |
| Bias | 9 | Describe any efforts to address potential sources of bias | ✓ page 7 |
| Study size | 10 | Explain how the study size was arrived at | ✓ pages 6-7 |
| Quantitative variables | 11 | Explain how quantitative variables were handled in the analyses. If applicable, describe which groupings were chosen and why | ✓ page 6-8 |
| Statistical methods | 12 | (*a*) Describe all statistical methods, including those used to control for confounding | ✓ page 7-8 |
| | | (*b*) Describe any methods used to examine subgroups and interactions | ✓ page 7-8 |
| | | (*c*) Explain how missing data were addressed | ✓ page 6 |
| | | (*d*) *Cohort study*—If applicable, explain how loss to follow-up was addressed<br>*Case-control study*—If applicable, explain how matching of cases and controls was addressed<br>*Cross-sectional study*—If applicable, describe analytical methods | ✓ page 7 |

For peer review only

| | | | |
|---|---|---|---|
| | | taking account of sampling strategy | |
| | | (*e*) Describe any sensitivity analyses | ✓ pages 7-8 |

Continued on next page

| | | | |
|---|---|---|---|
| **Results** | | | |
| Participants | 13* | (a) Report numbers of individuals at each stage of study—eg numbers potentially eligible, examined for eligibility, confirmed eligible, included in the study, completing follow-up, and analysed | ✓ pages 6,7 |
| | | (b) Give reasons for non-participation at each stage | ✓ page 6 |
| | | (c) Consider use of a flow diagram | n/a |
| Descriptive data | 14* | (a) Give characteristics of study participants (eg demographic, clinical, social) and information on exposures and potential confounders | ✓ Table 1 |
| | | (b) Indicate number of participants with missing data for each variable of interest | ✓Table 1 |
| | | (c) *Cohort study*—Summarise follow-up time (eg, average and total amount) | |
| Outcome data | 15* | *Cohort study*—Report numbers of outcome events or summary measures over time | |
| | | *Case-control study*—Report numbers in each exposure category, or summary measures of exposure | |
| | | *Cross-sectional study*—Report numbers of outcome events or summary measures | ✓pages 6,7 |
| Main results | 16 | (*a*) Give unadjusted estimates and, if applicable, confounder-adjusted estimates and their precision (eg, 95% confidence interval). Make clear which confounders were adjusted for and why they were included | ✓table 2 |
| | | (*b*) Report category boundaries when continuous variables were categorized | ✓ pages 6,7 |
| | | (*c*) If relevant, consider translating estimates of relative risk into absolute risk for a meaningful time period | |
| Other analyses | 17 | Report other analyses done—eg analyses of subgroups and interactions, and sensitivity analyses | ✓tables 3, 4 |
| **Discussion** | | | |
| Key results | 18 | Summarise key results with reference to study objectives | ✓ page 15 |
| Limitations | 19 | Discuss limitations of the study, taking into account sources of potential bias or imprecision. Discuss both direction and magnitude of any potential bias | ✓page 17 |
| Interpretation | 20 | Give a cautious overall interpretation of results considering objectives, limitations, multiplicity of analyses, results from similar studies, and other relevant evidence | ✓ pages 15-17 |
| Generalisability | 21 | Discuss the generalisability (external validity) of the study results | ✓ page 15-17 |
| **Other information** | | | |
| Funding | 22 | Give the source of funding and the role of the funders for the present study and, if applicable, for the original study on which the present article is based | ✓page 18 |

*Give information separately for cases and controls in case-control studies and, if applicable, for exposed and unexposed groups in cohort and cross-sectional studies.

For peer review only

**Note:** An Explanation and Elaboration article discusses each checklist item and gives methodological background and published examples of transparent reporting. The STROBE checklist is best used in conjunction with this article (freely available on the Web sites of PLoS Medicine at http://www.plosmedicine.org/, Annals of Internal Medicine at http://www.annals.org/, and Epidemiology at http://www.epidem.com/). Information on the STROBE Initiative is available at www.strobe-statement.org.

For peer review only



For peer review only - http://bmjopen.bmj.com/site/about/guidelines.xhtml

BMJ Open

# Ethnic differences in current smoking and former smoking in the Netherlands and the contribution of socio-economic factors: a cross-sectional analysis of the HELIUS Study

| | |
|---|---|
| Journal: | *BMJ Open* |
| Manuscript ID | bmjopen-2017-016041.R1 |
| Article Type: | Research |
| Date Submitted by the Author: | 17-Apr-2017 |
| Complete List of Authors: | Brathwaite, Rachel; London School of Hygiene and Tropical Medicine, Non-communicable Disease Epidemiology<br>Smeeth, Liam; London School of Hygiene and Tropical Medicine, Epidemiology and Population Health<br>Addo, Juliet; London School of Hygiene and Tropical Medicine, Epidemiology and Population Health<br>Kunst, Anton; University of Amsterdam, Dept. of Public Health, Academic Medical Center (AMC)<br>Peters, Ron; Academic Medical Center, Cardiology<br>Snijder, Marieke; Academic Medical Centre, University of Amsterdam, Department of Public Health<br>Derks, Eske; AMC, Department of Psychiatry; QIMR Berghofer , Translational Neurogenomics Group<br>Agyemang, Charles; Amsterdam Medical Center |
| <b>Primary Subject Heading</b>: | Smoking and tobacco |
| Secondary Subject Heading: | Smoking and tobacco, Epidemiology |
| Keywords: | EPIDEMIOLOGY, PUBLIC HEALTH, STATISTICS & RESEARCH METHODS |
| | |

SCHOLARONE™
Manuscripts

## TITLE PAGE

**Title:** Ethnic differences in current smoking and former smoking in the Netherlands and the contribution of socio-economic factors: a cross-sectional analysis of the HELIUS Study

**Corresponding Author:**

Rachel Brathwaite, PhD

Department of Non-communicable Disease Epidemiology, London School of Hygiene and Tropical Medicine, London, WC1E7HT, UK.

E-mail: Rachel.Brathwaite@lshtm.ac.uk

Telephone: +44 (0) 2076368636 ext 2495

**Authors:**

Rachel Brathwaite PhD[1], Liam Smeeth PhD[1], Juliet Addo PhD[1], Anton E Kunst PhD[2], Ron JG Peters PhD[3], Marieke B Snijder PhD[2], Eske M Derks PhD[4,5], Charles Agyemang PhD[2]

[1]Department of Non-Communicable Disease Epidemiology, London School of Hygiene and Tropical Medicine, London, UK.

[2]Department of Public Health, Academic Medical Center, University of Amsterdam, Amsterdam, The Netherlands.

[3]Department of Cardiology, Academic Medical Center, University of Amsterdam, Amsterdam, The Netherlands.

[4]Department of Psychiatry, Academic Medical Center, Amsterdam, The Netherlands.

[5]QIMR Berghofer, Translational Neurogenomics group, Brisbane, Australia

**Key words:** HELIUS study, smoking cessation, prevalence, ethnicity, socio-economic status (SES)

**Word count:** 3748



For peer review only

For peer review only - http://bmjopen.bmj.com/site/about/guidelines.xhtml

## ABSTRACT

**Objectives:** Data exploring how much of the ethnic differences in smoking prevalence and former smoking are explained by socioeconomic status (SES) are lacking. We therefore assessed ethnic differences in smoking prevalence and former smoking and the contribution of both educational level and occupational-related SES to the observed ethnic differences in smoking behaviour.

**Methods:** Data of 22,929 participants (aged 18-70 years) from the multi-ethnic cross-sectional HELIUS study in the Netherlands were analysed. Poisson regression models with a robust variance were used to estimate prevalence ratios.

**Results:** Compared to the Dutch, after adjustment for age and marital status, smoking prevalence was higher in men of Turkish (PR 1.69, 95% CI 1.54 to 1.86), African Surinamese (1.55, 1.41 to 1.69) and South-Asian Surinamese origin (1.53, 1.40 to 1.68), whereas among women, smoking prevalence was higher in Turkish, similar in African-Surinamese but lower in all other ethnic origin groups. All ethnic minority groups, except Ghanaians, had a significantly lower smoking cessation prevalence than the Dutch. Socio-economic gradients in smoking (higher prevalence among those lower educated and with lower level employment) were observed in all groups except Ghanaian women (a higher prevalence was observed in the higher educated). Ethnic differences in smoking prevalence and former smoking are largely, but not completely, explained by socioeconomic factors.

**Conclusions:** Our findings imply that anti-smoking policies designed to target smoking within the lower socioeconomic groups of ethnic minority populations may substantially reduce ethnic inequalities in smoking particularly among men and that certain groups may benefit from targeted smoking cessation interventions.


For peer review only

**Strengths and limitations**

- The HELIUS study utilized very large sample sizes in each ethnic group.
- Participants of the different ethnic groups were recruited in a systematic manner to reflect a representative sample of adults in each ethnic group living in Amsterdam.
- The same methodology was used to recruit participants from the different groups.
- Smoking status may be underreported since biochemical methods were not used to verify self-reported smoking statuses.
- Smoking cessation was assessed by using quit ratios, which is a cumulative measure of a number of former smokers in the ethnic group, which occurred over time and not necessarily an indicator of a recent smoking cessation or assessment at the individual level.

For peer review only



For peer review only - http://bmjopen.bmj.com/site/about/guidelines.xhtml

**WHAT IS ALREADY KNOWN ON THIS SUBJECT?**

In the Netherlands, ethnic differences in smoking has been observed among the Turkish, Surinamese and Moroccan ethnic minority groups with smoking being associated with most lower educated men and higher educated women. However, this was only investigated in the 35-60 age group, without distinguishing between African-Surinamese and South-Asian Surinamese and without including adults of Ghanaian descent, an African ethnic minority group in the Netherlands. There is limited recent data among Turkish and Moroccan groups which are important large migrant groups in several European countries. Comparisons of both smoking prevalence and former smoking has not been compared to the Dutch host population and the extent to which educational and occupational levels explain ethnic differences in smoking and former smoking has not been studied.

**WHAT DOES THIS STUDY ADD?**

This study compared smoking prevalence and former smoking of five large ethnic minority populations to the Dutch host population. The findings suggest that ethnic differences in smoking prevalence and former smoking in the Netherlands are largely but not completely explained by socio-economic differences. These findings imply that anti-smoking policies designed to target different ethnic groups and those of lower socio-economic status may substantially reduce smoking particularly among men in the Netherlands. However, among Ghanaian women, smoking prevention measures should particularly include higher educated women.



For peer review only - http://bmjopen.bmj.com/site/about/guidelines.xhtml

For peer review only

## INTRODUCTION

Ethnic differences in mortality rates and prevalence of lifestyle risk factors for diseases have been observed in the Netherlands.[1] Smoking is a leading lifestyle risk factor that accounts for premature morbidity and mortality from several diseases in populations worldwide.[2] Higher rates of smoking, as well as lower rates of former smoking are more present in disadvantaged than advantaged groups in society.[3-5] Most ethnic minority groups may be of lower socio-economic status (SES) than European host populations.[6] Evidence suggests variations in smoking prevalence across ethnic groups in high-income countries.[7,8] Differences in smoking prevalence among ethnic minority groups in European countries may be a reflection of their pre-migratory smoking status, or factors of the post-migratory environment, which influences smoking behaviour.[9,10] Smoking is associated with socio-economic,[11] social, and cultural factors which undergo changes following migration to new environments. In the Netherlands, researchers observed socio-economic gradients in smoking (smoking prevalence is higher as you have a lower socioeconomic group) among some ethnic minority populations, but not in sub-Saharan ethnic groups.[11] Inverse socio-economic gradients in smoking cessation (smoking cessation rate is lower as you have a lower socio-economic group) were recognised in other research.[12] Lower SES is associated with higher smoking possibly through mechanisms that include using smoking as a coping mechanism for stressful lives,[13,14] and less access to more costly and more effective options for smoking cessation.[15,16]

Few studies examined how much these differences in smoking and former smoking among ethnic minority groups relative to the Dutch were explained by socio-economic factors using both educational level and occupational level as socioeconomic indicators.[11,17]

In addition to ethnic differences in smoking, there are gender differences in tobacco usage, smoking behaviour,[18] acceptability of smoking, self-reporting of smoking, smoking cessation and factors that may influence the continuation of smoking globally.[19] It is well documented that men have higher smoking rates and higher daily cigarette consumption compared to women but this gender gap may vary among different ethnic groups and populations. We aimed to assess ethnic differences in smoking prevalence and former smoking among the Dutch, Ghanaian, Moroccan, Turkish, African Surinamese and South-Asian Surinamese origin ethnic groups in the Netherlands, and to study to what extent these ethnic differences could be possibly



For peer review only - http://bmjopen.bmj.com/site/about/guidelines.xhtml

For peer review only

explained by differences in SES. In order to be able to address this general aim, we checked whether smoking prevalence and former smoking was related to low SES not only among those of Dutch origin, but also among the ethnic minority groups. We also calculated age-standardized prevalences of current smoking and former smoking by ethnic group and gender, and compared differences in smoking prevalence and former smoking in each minority ethnic group relative to the Dutch host population.

## METHODS

### Study design and setting

Baseline data from the multi-ethnic HELIUS (**H**ealthy **Li**fe in an **U**rban **S**etting) study were used. Ethical approval was granted by the Institutional Review Board of the Academic Medical Center Amsterdam, and all participants provided written informed consent. In brief, HELIUS is investigating the patterns of health and health care utilization among the largest ethnic groups resident in Amsterdam, the Netherlands, including the Dutch host population and the ethnic minority groups of Ghanaian, Moroccan, Turkish, South-Asian Surinamese and African Surinamese origin.[20]

### Participants

Participants aged 18 to 70 years were randomly sampled from the Amsterdam municipal population register, stratified by ethnicity. Participants' ethnicity was defined according to the country of birth of the participant as well as that of his/her parents, which is currently the most widely accepted and most valid assessment of ethnicity in the Netherlands.[21] Specifically, a participant was considered as of ethnic minority origin if he/she fulfilled either of the following criteria: 1) he/she was born abroad and has at least one parent born abroad (first generation); or 2) he/she was born in the Netherlands but both his/her parents were born abroad (second generation). The Surinamese group was further classified according to self-reported ethnic origin into 'African', 'South-Asian', or other. Participants were considered of Dutch ethnic origin (henceforth, Dutch) if the participant and both parents were born in the Netherlands. Data for the HELIUS study were collected via the use of a questionnaire and a physical examination from January 2011 up to November 2015. For the current study, we used data of 23,942 participants for whom questionnaire data were available. Participants with an unknown/other ethnicity (n=50), Javanese Surinamese (n=250) or other/unknown Surinamese ethnicity



For peer review only - http://bmjopen.bmj.com/site/about/guidelines.xhtml

(n=286), and those with missing data on smoking status (n=107), marital status (n=134), and/or educational level (n=186) were excluded, resulting in a total sample size of 22,929 participants which was used in the current analyses.

**Smoking variables**

Smoking status was determined by the question 'Do you smoke at all?' Current smoking prevalence was calculated as the percentage of individuals in each ethnic group who responded 'yes' to this question. The prevalence of former smoking, also known as quit ratio percent,[22] in each ethnic group was determined by dividing the number of ex-smokers, (those who responded 'No, but I used to smoke') by the number of ever-smokers (the sum of ex and current smokers).

**Socioeconomic status variables**

The highest level of educational qualification attained (either in the Netherlands or in the country of origin) was grouped into three categories: 1) never been to school, or had elementary schooling only, or lower vocational schooling, or lower secondary schooling; 2) intermediate vocational schooling or higher secondary education schooling and; 3) higher vocational schooling or university level education. Occupational level was classified according to Dutch Standard Occupational Classification system for 2010.[23] This document provides an extensive systematic list of all professions in the Dutch system. Based on this document, occupational level was classified into 'elementary', 'lower', 'intermediate', 'higher', or 'graduate', based on job title and job description, including a question on fulfilling an executive function. Occupation-related SES was created by combining employment status (unemployed, not in working force (retired, student or full-time homeworker), incapacitated, or employed) with occupational level (elementary, lower, middle, higher or graduate level) among those who were employed. This resulted in the following categories: 1) unemployed, 2) not in the workforce, 3) incapacitated 4) employed at elementary/lower occupational level, and 5) employed at intermediate to graduate occupational level. The sixth category represented participants who were employed but there was insufficient data to define their occupational level. We grouped the elementary to lower and intermediate to graduate level categories of the occupation-related SES variable, due to small numbers in these categories across ethnic groups.



For peer review only - http://bmjopen.bmj.com/site/about/guidelines.xhtml

**Statistical methods**

Men and women were analysed separately since literature shows that smoking behaviour, including the usage and reasons for using tobacco and cessation, differs substantially by gender.[24] To make fair comparisons of the smoking prevalence across all ethnic groups, age-standardized prevalence rates for current smoking and former smoking were calculated using the entire HELIUS population as the standard population, stratified by 10-year age groups. A likelihood ratio test was used to test whether there were interactions between ethnicity and educational level and between ethnicity and occupation-related SES.

A Poisson regression model with a robust variance estimator was used to directly estimate adjusted prevalence ratios with 95% confidence intervals for current smoking and former smoking in each ethnic minority group relative to the Dutch.[25] Prevalence ratios have been widely recommended in the literature as the preferred estimate if the outcome of interest is common as odds ratio tends to greatly overestimate the risk ratio in cross-sectional studies.[25,26]

The SES of ethnic minority groups living in high-income countries may be lower than the native host population and the relationship between SES and risk factors for health is skewed in that lower SES groups have increased risk factor tendencies and worse health outcomes than the more economically advantaged groups.[27] Since we anticipated that SES may lie on the causal pathway between ethnicity and smoking behaviour,[20] we expect that the relationship between ethnicity and smoking will be mediated by SES. Therefore, we assessed the extent to which socio-economic factors explained ethnic variations in current smoking and former smoking by adjusting ethnic differences in smoking and former smoking for educational level and occupation-related SES along with age and marital status. This was compared to the models adjusted for age and marital status only.

The presence of significant interactions between ethnicity and educational level ($p<0.0001$) and between ethnicity and occupation-related SES ($p<0.0001$) in both genders overall were observed. We therefore further stratified the analysis by ethnic group and gender to assess the relationship between SES and smoking prevalence and former smoking while adjusting for age and marital status. All analyses were performed using STATA Version 14.1 and bar charts were plotted using Excel 2013.



For peer review only - http://bmjopen.bmj.com/site/about/guidelines.xhtml

## RESULTS

### Socio-demographic characteristics of study population

Table 1 shows the characteristics of the study population by gender and ethnic groups. On average, Moroccan and Turkish participants were younger than the Dutch, Ghanaian and Surinamese groups. Dutch men and women were more highly educated and had the lowest proportion of unemployed participants as compared with the ethnic minority groups.

Overall, the majority of ethnic minority groups were first generation migrants (76.1%), and 23.9% were second generation migrants. Ghanaians had the least amount of second generation migrants (5.3%), followed by African Surinamese (17.4%) and South-Asian Surinamese (24.6%) while the Turkish (31.5%) and Moroccan (33.5%) groups comprised of more second generation migrants.

### Current smoking

Among men, smoking was more common in those of Turkish, African Surinamese and South-Asian Surinamese origin than Moroccan and Dutch origin while Ghanaians smoked the least (Figure 1). Among women, fewer Ghanaian and Moroccan women smoked compared to Dutch, Turkish, African Surinamese and South-Asian Surinamese women (Figure 1). After adjustment for age and marital status, men of Turkish, African Surinamese and South-Asian Surinamese origin were significantly more likely than men of Dutch origin to be current smokers, whereas Moroccan men did not significantly differ from the Dutch (Table 2, model 1). Additional adjustment for SES variables reduced the increased prevalence ratio of current smoking observed among Turkish, South-Asian Surinamese and African-Surinamese men relative to Dutch men, however, they still smoked significantly more than the Dutch (Table 2, models 4). The direction of the association changed for Moroccan men from 10% more likely to 18% less likely to smoke than Dutch men and the likelihood that Ghanaian men were less likely to smoke increased from 74% to 81% less likely (95% CI 85% to 75%) than Dutch men to be smokers.

Among women, only Turkish women were significantly more likely to be current smokers as compared with Dutch women, whereas Moroccan, South-Asian Surinamese and Ghanaian women were significantly less likely



For peer review only - http://bmjopen.bmj.com/site/about/guidelines.xhtml

to be current smokers than women of Dutch origin (Table 2, model 1). For women, after additional adjustment for SES, women from all ethnic minority groups except Turkish were significantly less likely to smoke compared with Dutch women. Turkish women did not significantly differ from Dutch women anymore, after adjustment for SES (Table 2, models 4), whereas adjustment for SES made African-Surinamese women significantly less likely to be smokers than Dutch women.

For peer review only



For peer review only - http://bmjopen.bmj.com/site/about/guidelines.xhtml

Table 1: Socio-economic characteristics of the study population by ethnicity and sex

| | Dutch | Moroccan | Turkish | African Surinamese | South-Asian Surinamese | Ghanaian |
|---|---|---|---|---|---|---|
| **TOTAL SAMPLE SIZE N=22929 (%)** | **N=4598 (20.1%)** | **N=4262 (18.6%)** | **N=3985 (17.4%)** | **N=4369 (19.1%)** | **N=3320 (14.5%)** | **N=2395 (10.5%)** |
| **MEN N=9745** | **N=2115** | **N=1601** | **N=1800** | **N=1764** | **N=1536** | **N=929** |
| **Age, mean (SD)** | 46.9 (13.8) | 41.5 (12.9) | 40.2 (12.4) | 47.5 (13.4) | 44.3 (13.8) | 46.2 (12.0) |
| **Education, n (%)** | | | | | | |
| Higher vocational/ university | 1257 (59.4) | 297 (18.6) | 278 (15.4) | 312 (17.7) | 360 (23.4) | 85 (9.2) |
| Intermediate vocational/ higher secondary | 497 (23.5) | 539 (33.7) | 527 (29.3) | 608 (34.5) | 484 (31.5) | 283 (30.5) |
| Lower vocational or secondary and below | 361 (17.1) | 765 (47.8) | 995 (55.3) | 844 (47.9) | 692 (45.1) | 561 (60.4) |
| **Occupation, n (%)** | | | | | | |
| Unemployed | 124 (5.9) | 271 (17.0) | 285 (16.1) | 323 (18.5) | 227 (15.0) | 158 (16.1) |
| Not in the working force$^{\pi}$ | 338 (16.0) | 127 (8.0) | 129 (7.3) | 195 (11.2) | 180 (11.9) | 66 (7.2) |
| Incapacitated$^{\beta}$ | 57 (2.7) | 166 (10.4) | 158 (8.9) | 132 (7.6) | 126 (8.3) | 51 (5.5) |
| Employed at elementary/lower occupational level | 186 (8.8) | 515 (32.3) | 681 (38.3) | 529 (30.4) | 389 (25.6) | 517 (56.1) |
| Employed at middle to graduate occupational level | 1370 (64.8) | 452 (28.4) | 462 (26.0) | 510 (29.3) | 555 (36.6) | 94 (10.2) |
| Employed but unknown occupational level* | 38 (1.8) | 63 (4.0) | 61 (3.4) | 54 (3.1) | 41 (2.7) | 35 (3.8) |
| **WOMEN N=13184** | **N=2483** | **N=2661** | **N=2185** | **N=2605** | **N=1784** | **N=1466** |
| **Age, mean (SD)** | 45.4 (14.2) | 38.6 (13.0) | 39.5 (12.4) | 47.5 (12.4) | 45.7 (13.3) | 42.9 (11.0) |
| **Education, n (%)** | | | | | | |
| Higher vocational/ university | 1522 (61.3) | 439 (16.5) | 304 (13.9) | 672 (25.8) | 390 (21.9) | 66 (4.5) |
| Intermediate vocational/ higher secondary | 516 (20.8) | 633 (29.0) | 923 (34.7) | 958 (36.8) | 513 (28.8) | 333 (22.7) |
| Lower vocational or secondary and below | 445 (17.9) | 1299 (48.8) | 1248 (57.1) | 975 (37.4) | 881 (49.4) | 1067 (72.8) |
| **Occupation, n (%)** | | | | | | |
| Unemployed | 130 (5.3) | 389 (14.8) | 300 (13.9) | 385 (14.9) | 277 (15.7) | 408 (28.4) |
| Not in the working force$^{\pi}$ | 465 (18.8) | 1048 (39.9) | 792 (36.8) | 323 (12.5) | 297 (16.8) | 115 (8.0) |
| Incapacitated$^{\beta}$ | 84 (3.4) | 177 (6.7) | 199 (9.2) | 267 (10.3) | 179 (10.1) | 161 (11.2) |
| Employed at elementary/lower occupational level | 240 (9.7) | 349 (13.3) | 380 (17.6) | 417 (16.1) | 371 (21.0) | 611 (42.6) |
| Employed at middle to graduate occupational level | 1532 (61.9) | 607 (23.1) | 615 (34.8) | 1121 (43.4) | 615 (34.8) | 104 (7.3) |
| Employed but unknown occupational level* | 25 (1.0) | 59 (2.2) | 31 (1.8) | 73 (2.8) | 31 (1.8) | 36 (2.5) |

*working but did not provide sufficient information when describing their jobs; $^{\pi}$ retired or student or full-time homeworker; $^{\beta}$ disabled or unfit for work

For peer review only

Table 2: Prevalence and Adjusted Prevalence Ratios (PRs) with 95% CIs of current smoking of ethnic minority groups compared with Dutch

| Ethnic group | | Model 0 | Model 1* | Model 2** | Model 3*** | Model 4**** |
|---|---|---|---|---|---|---|
| | Crude Prevalence n/N (%) | PR (95% CI) | PR (95% CI) | PR(95% CI) | PR(95% CI) | PR (95% CI) |
| Men | | | | | | |
| Dutch | 562/2115 (26.6) | 1.00 | 1.00 | 1.00 | 1.00 | 1.00 |
| Turkish | 772/1800 (42.9) | 1.57 (1.44, 1.72) | 1.69 (1.54, 1.86) | 1.33 (1.20, 1.46) | 1.33 (1.20, 1.47) | 1.23 (1.11, 1.36) |
| African Surinamese | 780/1764 (44.2) | 1.68 (1.54, 1.84) | 1.55 (1.41, 1.69) | 1.26 (1.15, 1.39) | 1.28 (1.17, 1.41) | 1.20 (1.09, 1.32) |
| South-Asian Surinamese | 645/1536 (42.0) | 1.57 (1.43, 1.72) | 1.53 (1.40, 1.68) | 1.27 (1.15, 1.39) | 1.29 (1.17, 1.42) | 1.21 (1.10, 1.33) |
| Moroccan | 446/1601 (27.9) | 1.02 (0.92, 1.14) | 1.10 (0.99, 1.23) | 0.88 (0.79, 0.99) | 0.87 (0.78, 0.98) | 0.82 (0.73, 0.92) |
| Ghanaian | 69/929 (7.4) | 0.28 (0.22, 0.36) | 0.26 (0.20, 0.33) | 0.20 (0.16, 0.25) | 0.20 (0.16, 0.26) | 0.19 (0.15, 0.24) |
| Women | | | | | | |
| Dutch | 593/2483 (23.9) | 1.00 | 1.00 | 1.00 | 1.00 | 1.00 |
| Turkish | 647/2185 (29.6) | 1.15 (1.05, 1.27) | 1.26 (1.14, 1.40) | 1.07 (0.96, 1.20) | 1.09 (0.98, 1.21) | 1.03 (0.92, 1.15) |
| African Surinamese | 660/2605 (25.3) | 1.09 (0.99, 1.21) | 0.95 (0.86, 1.05) | 0.79 (0.71, 0.88) | 0.81 (0.74, 0.90) | 0.75 (0.68, 0.83) |
| South-Asian Surinamese | 370/1784 (20.7) | 0.88 (0.78, 0.98) | 0.82 (0.73, 0.92) | 0.68 (0.61, 0.77) | 0.70 (0.62, 0.79) | 0.65 (0.58, 0.73) |
| Moroccan | 145/2661 (5.5) | 0.21 (0.18, 0.25) | 0.23 (0.19, 0.27) | 0.19 (0.16, 0.23) | 0.20 (0.16, 0.24) | 0.19 (0.16, 0.23) |
| Ghanaian | 37/1466 (2.5) | 0.10 (0.07, 0.14) | 0.09 (0.06, 0.12) | 0.07 (0.05, 1.20) | 0.07 (0.05, 0.10) | 0.06 (0.05, 0.09) |

Model 0: Adjusted for age only; *Model 1: Adjusted for age and marital status; **Model 2: Adjusted for age, marital status, and education; ***Model 3: adjusted for age, marital status, and occupation-related SES; ****Model 4: adjusted for age, marital status, education and occupational-related SES



For peer review only - http://bmjopen.bmj.com/site/about/guidelines.xhtml

**Table 3: Prevalence and Adjusted Prevalence Ratios (PRs) with 95% CIs of former smoking of ethnic minority groups compared with Dutch**

| | Former smoking/ Quit ratio percent | | | | | |
|---|---|---|---|---|---|---|
| **Ethnic group** | | **Model 0** | **Model 1*** | **Model 2**** | **Model 3***** | **Model 4****** |
| | **Crude Prevalence n/N (%)** | **PR (95% CI)** | **PR (95% CI)** | **PR(95% CI)** | **PR(95% CI)** | **PR (95% CI)** |
| **Men** | | | | | | |
| **Dutch** | 827/1389 (59.5) | 1.00 | 1.00 | 1.00 | 1.00 | 1.00 |
| Turkish | 442/1214 (36.4) | 0.70 (0.64, 0.76) | 0.67 (0.62, 0.74) | 0.76 (0.71, 0.85) | 0.78 (0.71, 0.85) | 0.82 (0.74, 0.90) |
| African Surinamese | 363/1143 (31.8) | 0.52 (0.47, 0.57) | 0.57 (0.52, 0.63) | 0.63 (0.57, 0.70) | 0.63 (0.57, 0.70) | 0.65 (0.59, 0.72) |
| South-Asian Surinamese | 239/884 (27.0) | 0.48 (0.43, 0.54) | 0.50 (0.45, 0.56) | 0.56 (0.50, 0.63) | 0.56 (0.50, 0.63) | 0.58 (0.51, 0.65) |
| Moroccan | 432/878 (49.2) | 0.90 (0.84, 0.98) | 0.88 (0.82, 0.95) | 1.00 (0.92, 1.09) | 1.00 (0.92, 1.09) | 1.06 (0.97, 1.16) |
| Ghanaian | 118/187 (63.1) | 1.02 (0.91, 1.15) | 1.08 (0.96, 1.22) | 1.23 (1.09, 1.40) | 1.23 (1.09, 1.40) | 1.34 (0.74, 0.90) |
| **Women** | | | | | | |
| **Dutch** | 918/1511 (60.8) | 1.00 | 1.00 | 1.00 | 1.00 | 1.00 |
| Turkish | 253/900 (28.1) | 0.54 (0.48, 0.61) | 0.54 (0.48, 0.61) | 0.66 (0.58, 0.74) | 0.60 (0.53, 0.67) | 0.66 (0.59, 0.75) |
| African Surinamese | 470/1130 (41.6) | 0.68 (0.63, 0.74) | 0.75 (0.69, 0.81) | 0.84 (0.77, 0.92) | 0.79 (0.72, 0.85) | 0.85 (0.78, 0.92) |
| South-Asian Surinamese | 193/563 (34.3) | 0.60 (0.53, 0.68) | 0.64 (0.56, 0.72) | 0.73 (0.64, 0.82) | 0.68 (0.60, 0.76) | 0.73 (0.65, 0.83) |
| Moroccan | 93/238 (39.1) | 0.83 (0.70, 0.97) | 0.87 (0.74, 1.03) | 1.00 (0.85, 1.18) | 0.94 (0.80, 1.11) | 1.01 (0.86, 1.19) |
| Ghanaian | 75/112 (67.0) | 1.12 (0.97, 1.29) | 1.24 (1.07, 1.43) | 1.53 (1.31, 1.77) | 1.43 (1.22, 1.67) | 1.59 (1.36, 1.86) |

Model 0: Adjusted for age only; *Model 1: Adjusted for age and marital status; **Model 2: Adjusted for age, marital status, and education; ***Model 3: adjusted for age, marital status, and occupation-related SES; ****Model 4: adjusted for age, marital status, education and occupational-related SES



For peer review only - http://bmjopen.bmj.com/site/about/guidelines.xhtml

**Former smoking**

Among both sexes, the highest prevalence of former smoking was in Ghanaians while the lowest was in African-Surinamese men and Turkish women (Figure 2). All ethnic minority groups were significantly less likely to quit smoking as compared with Dutch, except for Ghanaians who were more likely to have quit (although not statistically significant in men) (Table 3, models 1). For former smoking, although the Turkish, Surinamese and Moroccan ethnic groups appeared to be less likely to quit smoking than the Dutch, the lower likelihoods were partly explained by socio-economic factors (Table 3, model 4). Ghanaians were even more likely to have a higher former smoking rate than the Dutch after socio-economic factors were accounted for.

**The relationship between SES and smoking prevalence and former smoking within each ethnic group**

Within each ethnic group, we observed SES inequalities in smoking prevalence (Table 4). For both sexes, participants with higher educational levels were less likely to smoke than those with lower educational levels in all ethnic groups, although not always statistically significant. Although very wide confidence intervals were obtained for Ghanaian women, those with higher vocational schooling or university level education smoked more than women with little or no education.

Compared to those employed in middle to graduate level positions, all unemployed men smoked more in all ethnic groups. Although not always statistically significant all unemployed and lower level professional women except Ghanaians were more likely to smoke. Persons who were disabled or unfit for work (incapacitated) also had a higher prevalence of smoking in all ethnic groups, although not statistically significant.

Within each ethnic group, SES inequalities were observed in former smoking (Table 5). For both sexes, participants with higher levels of education were more likely to quit smoking although not significant for Moroccan men and women. Higher educated Ghanaian women seemed less likely to quit smoking yet this was not significant. For both sexes, unemployed participants were less likely to quit compared to the middle or graduate level. Participants employed in lower level professional jobs were less likely to quit than those employed at the middle or graduate level except for Ghanaian women who appeared to be more likely to quit. A similar lower likelihood of quitting was observed among the incapacitated with the exception of Moroccan women.



For peer review only - http://bmjopen.bmj.com/site/about/guidelines.xhtml

Table 4: Current smoking prevalence ratios (95% CI) among men and women of different educational levels and occupation-related socio-economic status within each ethnic group

| Indicator of socioeconomic status | Dutch | South-Asian Surinamese | African Surinamese | Turkish | Moroccan | Ghanaian |
|---|---|---|---|---|---|---|
| | PR (95% CI)* | PR (95% CI)* | PR (95% CI)* | PR (95% CI)* | PR (95% CI)* | PR (95% CI)* |
| **Men** | | | | | | |
| **Education** | | | | | | |
| Higher vocational school/ university | 0.57 (0.47, 0.69) | 0.44 (0.36, 0.53) | 0.65 (0.55, 0.78) | 0.50 (0.41, 0.62) | 0.62 (0.49, 0.80) | 0.28 (0.07, 1.19) |
| Intermediate vocational/ higher secondary | 0.57 (0.57, 0.87) | 0.69 (0.61, 0.80) | 0.92 (0.82, 1.03) | 0.83 (0.73, 0.94) | 0.83 (0.70, 0.99) | 0.78 (0.46, 1.33) |
| Lower vocational and below | 1.00 | 1.00 | 1.00 | 1.00 | 1.00 | 1.00 |
| **Occupation-related SES** | | | | | | |
| Unemployed | 1.91 (1.53, 2.38) | 1.69 (1.43, 1.99) | 1.58 (1.35, 1.85) | 1.32 (1.11, 1.56) | 1.81 (1.46, 2.26) | 5.10 (1.17, 22.30) |
| Not in the working force$^{\pi}$ | 1.11 (0.87, 1.42) | 0.99 (0.76, 1.29) | 1.22 (0.96, 1.54) | 0.73 (0.53, 1.02) | 0.60 (0.36, 1.01) | 2.37 (0.37, 15.33) |
| Incapacitated$^{\beta}$ | 1.58 (1.10, 2.30) | 1.30 (1.02, 1.65) | 1.48 (1.21, 1.81) | 1.11 (0.88, 1.41) | 1.49 (1.11, 2.00) | 4.34 (0.83, 22.77) |
| Employed at elementary/lower occupational level | 1.50 (1.20, 1.87) | 1.57 (1.34, 1.84) | 1.32 (1.13, 1.53) | 1.27 (1.10, 1.46) | 1.33 (1.07, 1.64) | 3.37 (0.79, 14.37) |
| Employed at middle to graduate occupational level | 1.00 | 1.00 | 1.00 | 1.00 | 1.00 | 1.00 |
| **Women** | | | | | | |
| **Education** | | | | | | |
| Higher vocational school/university | 0.39 (0.33, 0.46) | 0.61 (0.47, 0.80) | 0.58 (0.48, 0.70) | 0.72 (0.56, 0.91) | 0.87 (0.53, 1.45) | 6.02 (2.23, 16.25) |
| Intermediate vocational/ higher secondary | 0.60 (0.50, 0.73) | 0.87 (0.70, 1.08) | 0.75 (0.65, 0.87) | 1.01 (0.87, 1.17) | 0.90 (0.59, 1.37) | 0.56 (0.19, 1.62) |
| Lower vocational and below | 1.00 | 1.00 | 1.00 | 1.00 | 1.00 | 1.00 |
| **Occupation-related SES** | | | | | | |
| Unemployed | 1.27 (0.96, 1.67) | 1.66 (1.30, 2.12) | 1.54 (1.29, 1.84) | 1.16 (0.94, 1.43) | 1.42 (0.90, 2.22) | 0.70 (0.18, 2.68) |
| Not in the working force$^{\pi}$ | 1.09 (0.86, 1.38) | 1.04 (0.74, 1.45) | 1.11 (0.86, 1.45) | 1.03 (0.85, 1.25) | 0.86 (0.53, 1.39) | 1.44 (0.33, 6.25) |
| Incapacitated$^{\beta}$ | 1.38 (1.01, 1.90) | 1.82 (1.34, 2.47) | 1.47 (1.17, 1.84) | 1.22 (0.95, 1.56) | 1.40 (0.77, 2.57) | 0.76 (0.17, 3.49) |
| Employed at elementary/lower occupational level | 1.69 (1.39, 2.07) | 1.23 (0.94, 1.61) | 1.34 (1.11, 1.62) | 1.29 (1.06, 1.57) | 1.22 (0.75, 1.99) | 0.44 (0.12, 1.67) |
| Employed at middle to graduate occupational level | 1.00 | 1.00 | 1.00 | 1.00 | 1.00 | 1.00 |

-*PR Adjusted for age and marital status; $^{\pi}$ retired or student or full-time homeworker; $^{\beta}$ disabled or unfit for work



For peer review only - http://bmjopen.bmj.com/site/about/guidelines.xhtml

**Table 5: Former smoking prevalence ratios (95% CI) among men and women of different educational levels and occupation-related socio-economic status within each ethnic group**

| Indicator of socioeconomic status | Dutch | South-Asian Surinamese | African Surinamese | Turkish | Moroccan | Ghanaian |
|---|---|---|---|---|---|---|
| | PR (95% CI)* | PR (95% CI)* | PR (95% CI)* | PR (95% CI)* | PR (95% CI)* | PR (95% CI)* |
| **Men** | | | | | | |
| **Education** | | | | | | |
| Higher vocational school/ university | 1.28 (1.14, 1.45) | 1.84 (1.44, 2.37) | 1.38 (1.12, 1.69) | 1.54 (1.27, 1.87) | 1.16 (0.95, 1.41) | 1.38 (1.05, 1.82) |
| Intermediate vocational/ higher secondary | 1.19 (1.04, 1.37) | 1.09 (0.82, 1.44) | 0.97 (0.80, 1.19) | 1.12 (0.94, 1.34) | 1.02 (0.88, 1.19) | 1.09 (0.84, 1.41) |
| Lower vocational and below | 1.00 | 1.00 | 1.00 | 1.00 | 1.00 | 1.00 |
| **Occupation-related SES** | | | | | | |
| Unemployed | 0.71 (0.56, 0.89) | 0.59 (0.41, 0.86) | 0.58 (0.44, 0.76) | 0.85 (0.67, 1.08) | 0.77 (0.61, 0.96) | 0.66 (0.47, 0.91) |
| Not in the working force$^{\pi}$ | 1.02 (0.91, 1.14) | 0.91 (0.64, 1.31) | 0.81 (0.62, 1.07) | 0.89 (0.60, 1.32) | 0.99 (0.75, 1.29) | 0.65 (0.36, 1.15) |
| Incapacitated$^{\beta}$ | 0.83 (0.63, 1.09) | 0.79 (0.52, 1.17) | 0.68 (0.48, 0.97) | 0.96 (0.74, 1.23) | 0.88 (0.70, 1.09) | 0.77 (0.51, 1.18) |
| Employed at elementary/lower occupational level | 0.82 (0.69, 0.98) | 0.57 (0.43, 0.76) | 0.72 (0.58, 0.89) | 0.85 (0.70, 1.03) | 0.93 (0.78, 1.11) | 0.63 (0.47, 0.84) |
| Employed at middle to graduate occupational level | 1.00 | 1.00 | 1.00 | 1.00 | 1.00 | 1.00 |
| **Women** | | | | | | |
| **Education** | | | | | | |
| Higher vocational school/university | 1.71 (1.52, 1.93) | 1.99 (1.33, 2.31) | 1.31 (1.10, 1.55) | 1.73 (1.33, 2.25) | 1.09 (0.71, 1.66) | 0.72 (0.36, 1.41) |
| Intermediate vocational/ higher secondary | 1.41 (1.21, 1.62) | 1.75 (1.33, 2.31) | 1.18 (1.00, 1.38) | 1.21 (0.95, 1.56) | 1.12 (0.78, 1.61) | 1.28 (0.98, 1.65) |
| Lower vocational and below | 1.00 | 1.00 | 1.00 | 1.00 | 1.00 | 1.00 |
| **Occupation-related SES** | | | | | | |
| Unemployed | 0.89 (0.73, 1.08) | 0.62 (0.44, 0.88) | 0.73 (0.58, 0.92) | 0.66 (0.48, 0.91) | 0.97 (0.61, 1.53) | 0.95 (0.60, 1.51) |
| Not in the working force$^{\pi}$ | 0.92 (0.83, 1.03) | 0.77 (0.55, 1.07) | 0.98 (0.79, 1.20) | 0.54 (0.40, 0.73) | 1.02 (0.64, 1.62) | 0.52 (0.22, 1.22) |
| Incapacitated$^{\beta}$ | 0.86 (0.69, 1.08) | 0.57 (0.38, 0.87) | 0.85 (0.69, 1.05) | 0.65 (0.45, 0.96) | 1.20 (0.75, 1.91) | 0.97 (0.55, 1.71) |
| Employed at elementary/lower occupational level | 0.76 (0.65, 0.89) | 0.48 (0.32, 0.73) | 0.76 (0.61, 0.94) | 0.60 (0.43, 0.82) | 0.84 (0.49, 1.44) | 1.07 (0.69, 1.66) |
| Employed at middle to graduate occupational level | 1.00 | 1.00 | 1.00 | 1.00 | 1.00 | 1.00 |

-*PR Adjusted for age and marital status; $^{\pi}$ retired or student or full-time homeworker; $^{\beta}$ disabled or unfit for work



For peer review only - http://bmjopen.bmj.com/site/about/guidelines.xhtml

## DISCUSSION

### Key findings

The prevalence of current smoking and former smoking of adult men and women of minority ethnic groups living in Amsterdam is different from the Dutch host population. Accounting for socio-economic factors, the analysis showed that Turkish, South-Asian Surinamese and African Surinamese men smoked more than Dutch men while Ghanaian and Moroccan men smoked less. The smoking prevalence of Turkish women was similar to Dutch women while Ghanaian, Moroccan, South-Asian Surinamese and African Surinamese women smoked less. Only the Ghanaian ethnic group was more likely to quit than the Dutch. Socio-economic factors influenced current smoking and former smoking prevalence, but more so among men than among women and differently depending on the ethnic minority group. SES contributed to the higher prevalence of smoking among Turkish, Surinamese and Moroccan men and Turkish women, but diminished the estimates for Surinamese, Moroccan and Ghanaian women who were less likely to smoke than the Dutch. Socio-economic factors also accounted for part of the lower prevalence of former smoking observed among the ethnic minority groups relative to the Dutch.

### Discussion of key findings

Smoking behaviour differed among the various ethnic minority groups compared with the Dutch. The prevalence of smoking among Ghanaians in the Netherlands has not been previously studied. Comparable studies in Amsterdam, which used the same country of birth criteria for ethnicity, indicates that smoking prevalence among adults aged 35-60 years has reduced over the last decade (from 2000/2003 to 2011/2015) in Moroccan, Turkish, and Surinamese groups.[11] Age-standardized estimates of smoking prevalence among Turkish men (63%) declined to ~41.7%, and from 55% to 44.8% in Surinamese men, but remained fairly constant in Moroccan men (~29%). However, a slight increase in smoking prevalence from <1% to ~2.6% among Moroccan women was observed. The presence of stronger tobacco control policies in the Netherlands after 2001 may be a contributing factor for the decline in smoking prevalence among Turkish and Surinamese men over the last decade bringing it closer to Dutch men.[28]

When referring to estimates of smoking from population-based studies conducted in the respective countries of origin, we observed more variability between women in the home country vs women in Amsterdam: 13% in



For peer review only - http://bmjopen.bmj.com/site/about/guidelines.xhtml

Turkey vs 27% (Turkish),[29] 9.9% in Suriname vs 24.2% (Surinamese),[30] 3.3% in Morocco vs 4.3% (Moroccan),[31] and 0.3% in Ghana vs 2.9% (Ghanaian).[32] Whereas for men, closer parallels between men in their home countries and men in Amsterdam: 41% vs 40.5% (Turkish),[29] 38.4% vs 43.2% (Surinamese),[30] ; 31.5% vs 26.8% (Moroccan),[31]; and 8.9% vs 7.5% (Ghanaian) were detected.[32] These differences, especially among women, allude to the possible impact of acculturation, and different social norms on smoking.[8-10,33] Focusing targeted ethnic smoking interventions on people at the same time they immigrate maybe a potential strategy that can be used for smoking prevention and reduction.

From our findings, lower SES was related to higher smoking prevalence and lower former smoking more in men in most ethnic groups and is consistent with findings from past research.[34-36] Smoking was more common among lower educated men of Turkish and host populations in a study conducted in Germany and the Netherlands.[37] However, higher educated Turkish women smoked more than lower educated while the reverse was evident in the host population.[37] Higher smoking among the more educated women was observed only in Ghanaian women but very wide confidence intervals were obtained due to small numbers. Higher smoking among the more educated women in Amsterdam may flag the signs of the early stages of tobacco smoking epidemic among Ghanaian women,[38,39] who may be challenging cultural norms and taboos towards smoking present especially among women in the Ghanaian community.

For former smoking, higher prevalences among the Dutch than African and South-Asian Surinamese are known.[17] This study confirmed that Turkish and Surinamese smokers have lower former smoking prevalence while Ghanaian smokers are more likely to quit than the Dutch. There were indications that Moroccan and Dutch had similar quit ratios. Others factors including lack of social support, more stressful lives, or living in deprived neighbourhoods may be ways in which lower SES indirectly influences smoking cessation.[40]

Higher rates of smoking were observed among both men and women employed in lower level professional jobs than those in higher professional jobs of all ethnic groups except Ghanaian and Moroccan men in which the difference may not have been detected due to small numbers. Enforcing restrictions on smoking on the workplace can make it more difficult for even workers in lower level jobs to smoke on the job.[41]

Although SES contributed to observed ethnic differences in smoking and cessation, it did not entirely explain the ethnic differences suggesting that other factors may play a role. A possible explanation is that smoking



For peer review only - http://bmjopen.bmj.com/site/about/guidelines.xhtml

behaviour may be influenced by cultural norms present in the ethnic group, as those sharing a particular ethnic identity tend to share similar attitudes towards smoking.[42]

The strengths of the HELIUS study is that it utilized large sample sizes in each ethnic group and participants were recruited in a systematic manner to reflect a representative sample of adults in each ethnic group living in Amsterdam.[20] Smoking status may be underreported since biochemical methods were not used to verify self-reported smoking statuses. People tend to underreport undesirable health behaviours especially those stigmatized within the wider society.[43] Urine analysis of cotinine levels, the main metabolite of nicotine, is a useful biochemical indicator that can be used to validate current smoking among participants.[44] Our research did not focus on analysing what current smokers smoked, however the majority used cigarettes rather than cigars and packages of pipe tobacco. Smoking cessation was assessed by using quit ratios, which is a cumulative measure of a number of former smokers in the ethnic group, which occurred over time and not necessarily an indicator of a recent smoking cessation or assessment at the individual level.[41]

## CONCLUSION

Current smoking and former smoking varies by ethnic group in the Netherlands. The higher levels of smoking prevalence and the lower levels of former smoking among some ethnic minority groups compared to the Dutch are largely, but not completely, explained by socioeconomic factors. Although differences were less marked after adjustment, particularly for socioeconomic factors, the findings still suggest that some ethnic minority groups may benefit from targeted cessation interventions.



For peer review only - http://bmjopen.bmj.com/site/about/guidelines.xhtml

## FUNDING

The HELIUS study is funded by the Dutch Heart Foundation (Hartstichting) (grant number 2010T084), the Netherlands Organization for Health Research and Development (ZonMw grant number 200500003), and the European Commission Seventh Framework Programme (FP-7) (grant number 278901). The views expressed in the paper are those of the authors, the funders had no participation in the writing of this paper.

## DECLARATION OF INTETESTS

None declared.

## AUTHOR CONTRIBUTIONS

The study idea was conceived my RB in collaboration with LS, JA, and CA. The first draft of the paper was written by RB. LS, JA, CA, EMD, AEK, RP, MBS, all wrote and contributed to several updated versions.

## ACKNOWLEDGEMENTS

The HELIUS study is conducted by the Academic Medical Center Amsterdam and the Public Health Service of Amsterdam. Both organisations provided core support for HELIUS. We are most grateful to the participants of the HELIUS study and the management team, research nurses, interviewers, research assistants and other staff who have taken part in gathering the data of this study.

**Figure Legends**

Figure 1 Age-standardized prevalence of current smoking by gender and ethnicity

Figure 2 Age-standardized prevalence of former smoking by gender and ethnicity

For peer review only

## REFERENCES


1. Bos V, Kunst AE, Keij-Deerenberg IM, Garssen J, Mackenbach JP. Ethnic inequalities in age- and cause-specific mortality in the Netherlands. *Int. J. Epidemiol.* 2004;33(5):1112-1119 10.1093/ije/dyh189.
2. World Health Organization. *WHO REPORT ON THE GLOBAL TOBACCO EPIDEMIC 2008.* Geneva: World Health Organization;2008
3. ASH, Health Development Agency. Smoking and Health Inequalities. London.
4. Centers for Disease Control and Prevention. Cigarette smoking among adults-United States 1998. *Morbidity and Mortality Weekly Report.* 2000;49(39):881-884
5. Department of Health. Smoking kills A white paper on tobacco. London: The Stationery Office; 1998.
6. Bos V. *Ethnic inequalities in mortality in the Netherlands and the role of socioeconomic status*. Rotterdam, the Netherlands: Department of Public Health, Erasmus MC Univeristy Medical Centre; 2005.
7. Scarborough P, Bhatnagar P, Kaur A, et al. *Ethnic Differences in Cardiovascular Disease 2010 Edition.* British Heart Foundation Health Promotion Research Group,Department of Public Health, University of Oxford;2010
8. Wilkinson AV, Spitz MR, Strom SS, et al. Effects of nativity, age at migration, and acculturation on smoking among adult Houston residents of Mexican descent. *Am. J. Public Health.* 2005;95(6):1043-1049 10.2105/AJPH.2004.055319).
9. Bethel JW, Schenker MB. Acculturation and smoking patterns among Hispanics: A review. *Am. J. Prev. Med.* 2005;29(2):143-148 doi:10.1016/j.ampere.2005.04.014.
10. Choi S, Rankin S, Stewart A, Oka R. Effects of acculturation on Smoking Behavior in Asian Americans: A Meta-Analysis. *J. Cardiovasc. Nurs.* 2008;23(1):67-73 10.1097/1001.JCN.0000305057.0000396247.f0000305052 10.1097/01.JCN.0000305057.96247.f2.
11. Nierkens V, de Vries H, Stronks K. Smoking in immigrants: do socioeconomic gradients follow the pattern expected from the tobacco epidemic? *Tob. Control.* 2006;15(5):385-391 10.1136/tc.2006.014902.
12. Fernandez E, Schiaffino A, García M, Borràs JM. Widening social inequalities in smoking cessation in Spain, 1987–1997. *J. Epidemiol. Community Health.* 2001;55(10):729-730 10.1136/jech.55.10.729.
13. Lantz PM, House JS, Mero RP, Williams DR. Stress, life events, and socioeconomic disparities in health: results from the Americans' Changing Lives Study. *J. Health Soc. Behav.* 2005;46(3):274-288
14. Layte R, Whelan CT. Explaining Social Class Inequalities in Smoking: The Role of Education, Self-Efficacy, and Deprivation. *European Sociological Review.* 2009;25(4):399-410 10.1093/esr/jcn022.
15. Manley MW, Griffin T, Foldes SS, Link CC, Sechrist RA. The role of health plans in tobacco control. *Annu. Rev. Public Health.* 2003;24:247-266 10.1146/annurev.publhealth.24.100901.140838.
16. Bauer JE, Hyland A, Li Q, Steger C, Cummings KM. A longitudinal assessment of the impact of smoke-free worksite policies on tobacco use. *Am. J. Public Health.* 2005;95(6):1024-1029 10.2105/ajph.2004.048678.
17. Agyemang C, Stronks K, Tromp N, et al. A cross-national comparative study of smoking prevalence and cessation between English and Dutch South Asian and African origin populations: the role of national context. *Nicotine Tob. Res.* 2010;12(6):557-566 10.1093/ntr/ntq044.




For peer review only - http://bmjopen.bmj.com/site/about/guidelines.xhtml

18. Bauer T, Gohlmann S, Sinning M. Gender differences in smoking behavior. *Health Econ.* 2007;16(9):895-909 10.1002/hec.1259.
19. The Tobacco Atlas, Fifth Edition. American Cancer Society, Inc; 2015. http://www.tobaccoatlas.org/.
20. Stronks K, Snijder M, Peters R, et al. Unravelling the impact of ethnicity on health in Europe: the HELIUS study. *BMC Public Health.* 2013;13(1):1-10 10.1186/1471-2458-13-402.
21. Stronks K, Kulu-Glasgow I, Agyemang C. The utility of 'country of birth' for the classification of ethnic groups in health research: the Dutch experience. *Ethn. Health.* 2009;14(3):255-269 10.1080/13557850802509206.
22. Kulik MC, Glantz SA. The smoking population in the USA and EU is softening not hardening. *Tob. Control.* 2015 doi:10.1136/tobaccocontrol-2015-052329.
23. Central Bureau of Statistics. *Standard Occupational Classification 2010.* 2010
24. National Institute on Drug Abuse, National Institutes of Health, U.S. Department of Health and Human Services. *Tobacco/Nicotine.* 2016
25. Coutinho LM, Scazufca M, Menezes PR. Methods for estimating prevalence ratios in cross-sectional studies. *Revista de saude publica.* 2008;42(6):992-998 http://dx.doi.org/10.1590/S0034-89102008000600003
26. Barros A, Hirakata V. Alternatives for logistic regression in cross-sectional studies: an empirical comparison of models that directly estimate the prevalence ratio. *BMC Med. Res. Methodol.* 2003;3(1):1-13 10.1186/1471-2288-3-21.
27. Richard Wilkinson, Marmot M, eds. *Social determinants of health: the solid facts.* 2nd ed. Denmark: World Health Organisation; 2003.
28. Bosdriesz JR, Nagelhout GE, Stronks K, Willemsen MC, Kunst AE. The association between tobacco control policy and educational inequalities in smoking cessation in the Netherlands from 1988 through 2011. *Nicotine Tob. Res.* 2015 10.1093/ntr/ntv004.
29. Republic of Turkey Ministry of Health. *Global Adult Tobacco Survey Turkey 2012.* Ankara2014
30. World Health Organization. *WHO country report on the global tobacco epidemic, 2012 country profile Suriname.* 2013
31. Tachfouti N, El RK, Berraho M, et al. Knowledge and attitude about antismoking legislation in Morocco according to smoking status. *East. Mediterr. Health J.* 2011;17(4):297-302
32. Owusu-Dabo E, Lewis S, McNeill A, Gilmore A, Britton J. Smoking uptake and prevalence in Ghana. *Tob. Control.* 2009;18(5):365-370 http://dx.doi.org/10.1136/tc.2009.030635.
33. Perez-Stable EJ, Ramirez A, Villareal R, et al. Cigarette smoking behavior among US Latino men and women from different countries of origin. *Am. J. Public Health.* 2001;91(9):1424-1430
34. Cavelaars AEJM, Kunst AE, Geurts JJM, et al. *Educational differences in smoking: international comparison.* Vol 3202000.
35. Faggiano F, Versino E, Lemma P. Decennial trends of social differentials in smoking habits in Italy. *Cancer Causes Control.* 2001;12(7):665-671 10.1023/A:101124702497 9.
36. Galobardes B, Costanza MC, Bernstein MS, Delhumeau C, Morabia A. Trends in risk factors for lifestyle-related diseases by socioeconomic position in Geneva, Switzerland, 1993–2000: Health inequalities persist. *Am. J. Public Health.* 2003;93(8):1302-1309
37. Reiss K, Sauzet O, Breckenkamp J, Spallek J, Razum O. How immigrants adapt their smoking behaviour: comparative analysis among Turkish immigrants in Germany and the Netherlands. *BMC Public Health.* 2014;14:844 10.1186/1471-2458-14-844.
38. Pampel FC. Age and education patterns of smoking among women in high-income nations. *Soc. Sci. Med.* 2003;57(8):1505-1514 http://dx.doi.org/10.1016/S0277-9536(02)00543-9.
39. Pampel FC. Cigarette diffusion and sex differences in smoking. *J. Health Soc. Behav.* 2001;42(4):388-404

For peer review only



For peer review only - http://bmjopen.bmj.com/site/about/guidelines.xhtml

40. Businelle MS, Kendzor DE, Reitzel LR, et al. Mechanisms linking socioeconomic status to smoking cessation: a structural equation modeling approach. *Health Psychol.* 2010;29(3):262-273 10.1037/a0019285.
41. Burns DM. Smoking Cessation: Recent inidcators of what's working at a population level. *Smoking and Tobacco Control Monograph No. 12.* Vol 12: National Cancer Institute; 2000.
42. Nichter M. Smoking: what does culture have to do with it? *Addiction.* 2003;98 Suppl 1:139-145
43. Elkind AK. The social definition of women's smoking behaviour. *Soc. Sci. Med.* 1985;20(12):1269-1278 10.1016/0277-9536(85)90380-6.
44. Benowitz NL, Jacob P. Metabolism of nicotine to cotinine studied by a dual stable isotope method. *Clin. Pharmacol. Ther.* 1994;56(5):483-493 10.1038/clpt.1994.169.

For peer review only



For peer review only - http://bmjopen.bmj.com/site/about/guidelines.xhtml

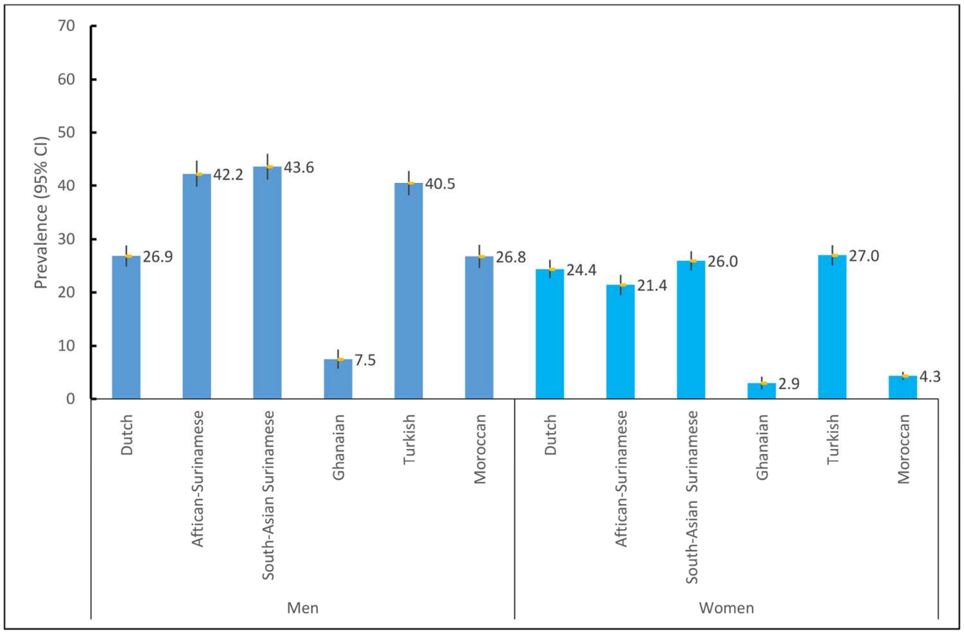


Figure 1 Age-standardized prevalence of current smoking by gender and ethnicity

165x107mm (300 x 300 DPI)

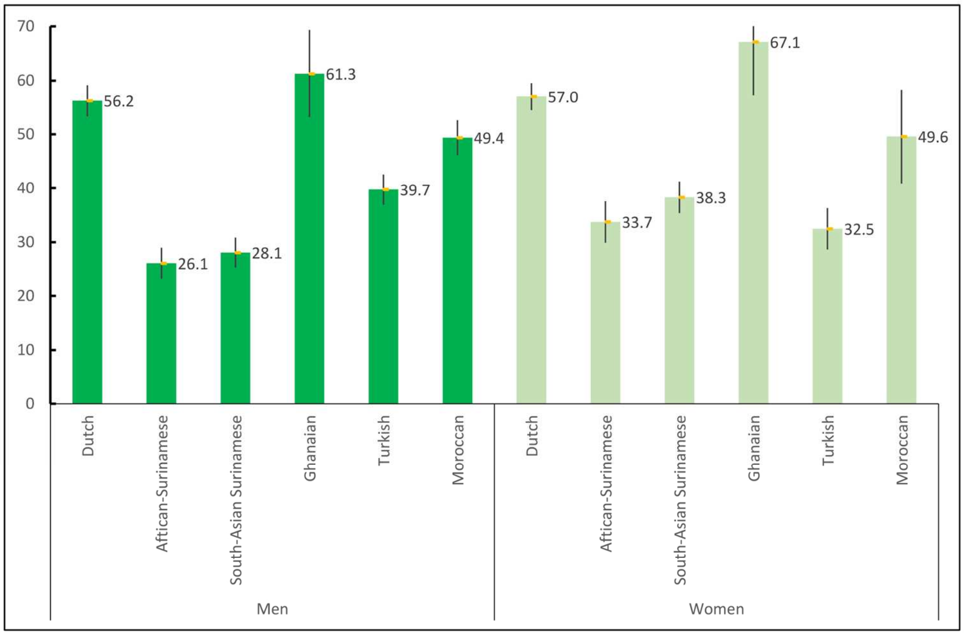


Figure 2 Age-standardized prevalence of former smoking by gender and ethnicity

165x107mm (300 x 300 DPI)

STROBE Statement—checklist of items that should be included in reports of observational studies

| | Item No | Recommendation | |
|---|---|---|---|
| **Title and abstract** | 1 | (*a*) Indicate the study's design with a commonly used term in the title or the abstract | ✓Abstract |
| | | (*b*) Provide in the abstract an informative and balanced summary of what was done and what was found | ✓ |
| **Introduction** | | | |
| Background/rationale | 2 | Explain the scientific background and rationale for the investigation being reported | ✓ page 5 |
| Objectives | 3 | State specific objectives, including any prespecified hypotheses | ✓ pages 5,6 |
| **Methods** | | | |
| Study design | 4 | Present key elements of study design early in the paper | ✓ page 6 |
| Setting | 5 | Describe the setting, locations, and relevant dates, including periods of recruitment, exposure, follow-up, and data collection | ✓ page 6 |
| Participants | 6 | (*a*) *Cohort study*—Give the eligibility criteria, and the sources and methods of selection of participants. Describe methods of follow-up<br>*Case-control study*—Give the eligibility criteria, and the sources and methods of case ascertainment and control selection. Give the rationale for the choice of cases and controls<br>*Cross-sectional study*—Give the eligibility criteria, and the sources and methods of selection of participants | ✓ pages 6,7 |
| | | (*b*) *Cohort study*—For matched studies, give matching criteria and number of exposed and unexposed<br>*Case-control study*—For matched studies, give matching criteria and the number of controls per case | |
| Variables | 7 | Clearly define all outcomes, exposures, predictors, potential confounders, and effect modifiers. Give diagnostic criteria, if applicable | ✓ page 7 |
| Data sources/ measurement | 8* | For each variable of interest, give sources of data and details of methods of assessment (measurement). Describe comparability of assessment methods if there is more than one group | ✓ page 7 |
| Bias | 9 | Describe any efforts to address potential sources of bias | ✓ page 7-8 |
| Study size | 10 | Explain how the study size was arrived at | ✓ pages 6-7 |
| Quantitative variables | 11 | Explain how quantitative variables were handled in the analyses. If applicable, describe which groupings were chosen and why | ✓ page 6-8 |
| Statistical methods | 12 | (*a*) Describe all statistical methods, including those used to control for confounding | ✓ page 7-8 |
| | | (*b*) Describe any methods used to examine subgroups and interactions | ✓ page 7-8 |
| | | (*c*) Explain how missing data were addressed | ✓ page 6-7 |
| | | (*d*) *Cohort study*—If applicable, explain how loss to follow-up was addressed<br>*Case-control study*—If applicable, explain how matching of cases and controls was addressed<br>*Cross-sectional study*—If applicable, describe analytical methods | ✓ page 7-8 |

For peer review only

| | | | |
|---|---|---|---|
| | | taking account of sampling strategy | |
| | | (*e*) Describe any sensitivity analyses | ✓ pages 7-8 |

Continued on next page

| Results | | | |
|---|---|---|---|
| Participants | 13* | (a) Report numbers of individuals at each stage of study—eg numbers potentially eligible, examined for eligibility, confirmed eligible, included in the study, completing follow-up, and analysed | ✓ pages 6,7 |
| | | (b) Give reasons for non-participation at each stage | ✓ page 6 |
| | | (c) Consider use of a flow diagram | n/a |
| Descriptive data | 14* | (a) Give characteristics of study participants (eg demographic, clinical, social) and information on exposures and potential confounders | ✓ Table 1 |
| | | (b) Indicate number of participants with missing data for each variable of interest | ✓Table 1 |
| | | (c) *Cohort study*—Summarise follow-up time (eg, average and total amount) | |
| Outcome data | 15* | *Cohort study*—Report numbers of outcome events or summary measures over time | |
| | | *Case-control study*—Report numbers in each exposure category, or summary measures of exposure | |
| | | *Cross-sectional study*—Report numbers of outcome events or summary measures | ✓pages 6,7 |
| Main results | 16 | (*a*) Give unadjusted estimates and, if applicable, confounder-adjusted estimates and their precision (eg, 95% confidence interval). Make clear which confounders were adjusted for and why they were included | ✓Tables 2 and 3 |
| | | (*b*) Report category boundaries when continuous variables were categorized | ✓ pages 6,7 |
| | | (*c*) If relevant, consider translating estimates of relative risk into absolute risk for a meaningful time period | |
| Other analyses | 17 | Report other analyses done—eg analyses of subgroups and interactions, and sensitivity analyses | ✓Tables 4 and 5 |

| Discussion | | | |
|---|---|---|---|
| Key results | 18 | Summarise key results with reference to study objectives | ✓ page 17 |
| Limitations | 19 | Discuss limitations of the study, taking into account sources of potential bias or imprecision. Discuss both direction and magnitude of any potential bias | ✓page 19 |
| Interpretation | 20 | Give a cautious overall interpretation of results considering objectives, limitations, multiplicity of analyses, results from similar studies, and other relevant evidence | ✓ pages 17-19 |
| Generalisability | 21 | Discuss the generalisability (external validity) of the study results | ✓ page 17-19 |

| Other information | | | |
|---|---|---|---|
| Funding | 22 | Give the source of funding and the role of the funders for the present study and, if applicable, for the original study on which the present article is based | ✓page 20 |

*Give information separately for cases and controls in case-control studies and, if applicable, for exposed and unexposed groups in cohort and cross-sectional studies.



For peer review only - http://bmjopen.bmj.com/site/about/guidelines.xhtml

**Note:** An Explanation and Elaboration article discusses each checklist item and gives methodological background and published examples of transparent reporting. The STROBE checklist is best used in conjunction with this article (freely available on the Web sites of PLoS Medicine at http://www.plosmedicine.org/, Annals of Internal Medicine at http://www.annals.org/, and Epidemiology at http://www.epidem.com/). Information on the STROBE Initiative is available at www.strobe-statement.org.

For peer review only



For peer review only - http://bmjopen.bmj.com/site/about/guidelines.xhtml
